# Early Apoptosis of Macrophages Modulated by Injection of *Yersinia pestis* YopK Promotes Progression of Primary Pneumonic Plague

**DOI:** 10.1371/journal.ppat.1003324

**Published:** 2013-04-25

**Authors:** Kristen N. Peters, Miqdad O. Dhariwala, Jennifer M. Hughes Hanks, Charles R. Brown, Deborah M. Anderson

**Affiliations:** 1 Department of Veterinary Pathobiology, University of Missouri, Columbia, Missouri, United States of America; 2 Laboratory for Infectious Disease Research, University of Missouri, Columbia, Missouri, United States of America; 3 Department of Molecular Microbiology & Immunology, University of Missouri, Columbia, Missouri, United States of America; Tufts University School of Medicine, United States of America

## Abstract

*Yersinia pestis* causes pneumonic plague, a disease characterized by inflammation, necrosis and rapid bacterial growth which together cause acute lung congestion and lethality. The bacterial type III secretion system (T3SS) injects 7 effector proteins into host cells and their combined activities are necessary to establish infection. *Y. pestis* infection of the lungs proceeds as a biphasic inflammatory response believed to be regulated through the control of apoptosis and pyroptosis by a single, well-conserved T3SS effector protein YopJ. Recently, YopJ-mediated pyroptosis, which proceeds via the NLRP3-inflammasome, was shown to be regulated by a second T3SS effector protein YopK in the related strain *Y. pseudotuberculosis*. In this work, we show that for *Y. pestis*, YopK appears to regulate YopJ-mediated apoptosis, rather than pyroptosis, of macrophages. Inhibition of caspase-8 blocked YopK-dependent apoptosis, suggesting the involvement of the extrinsic pathway, and appeared cell-type specific. However, in contrast to *yopJ*, deletion of *yopK* caused a large decrease in virulence in a mouse pneumonic plague model. YopK-dependent modulation of macrophage apoptosis was observed at 6 and 24 hours post-infection (HPI). When YopK was absent, decreased populations of macrophages and dendritic cells were seen in the lungs at 24 HPI and correlated with resolution rather than progression of inflammation. Together the data suggest that *Y. pestis* YopK may coordinate the inflammatory response during pneumonic plague through the regulation of apoptosis of immune cells.

## Author Summary

In this work, we studied the mechanism whereby bacteria manipulate innate immune responses by controlling host cell death. *Yersinia pestis*, the causative agent of plague, requires effector Yops of the Type III Secretion System (T3SS) to evade the innate immune system during infection. We show that *Yersinia* induces apoptosis of macrophages through two distinct mechanisms, each through the activity of the well-characterized T3SS effector YopJ, yet regulated in an opposing manner through the activity of a second effector protein YopK. In a murine pneumonic plague model, we found evidence that YopK regulates apoptosis of macrophages during the early stage of infection, leading to uncontrolled inflammation and disease. In contrast, the absence of YopK-regulated apoptosis allowed recruitment of lymphocytes and CCR2^+^ immune cells which led to bacterial clearance and resolution of inflammation. Together the data suggest that *Yersinia* YopK modulates apoptosis of immune cells to control the inflammatory response during plague.

## Introduction

Acute bacterial pneumonia is the result of active colonization of the airspace in the lungs combined with host inflammation that is unable to resolve due to host-pathogen interactions as well as progressing host- and microbial- induced injury. Resident macrophages in the lungs play an important role in orchestrating the mucosal immune response and subsequent tissue repair following infection [1]. Alveolar and interstitial macrophages act as sentinel cells and react to pathogen-associated molecular patterns following bacterial invasion of the lung mucosa by activating pro-inflammatory cytokine production and phagocytosis. Following chemotaxis, neutrophils are the primary mediators of bacterial clearance. After neutrophils destroy invading extracellular bacteria, interstitial macrophages activate a resolution program, allowing efferocytosis and clearance of apoptotic neutrophils [2]. Activated alveolar macrophages retain a pro-inflammatory role and apoptosis of these macrophages signals the down-regulation of inflammation and induction of tissue repair. When efferocytosis by interstitial macrophages does not occur, increased severity of pneumonia results while, conversely, treatment of mice with apoptotic macrophages is protective against lethality [3], [4]. Apoptosis provides both pro- and anti-inflammatory signals and each is necessary to prevent bacterial pneumonia.

*Yersinia pestis* is a Gram negative bacterium that causes bubonic, pneumonic, and septicemic plague [5]. Expression of multiple virulence factors together allow evasion and manipulation of the host innate immune system and rapid replication in the midst of a massive pro-inflammatory response [6]. Pulmonary infection of mammals progresses as an acute bronchopneumonia, with an initial delay in inflammatory responses thought to be important to successful infection [7], [8]. Near 48 hours post-infection (HPI), bacteria multiply rapidly and cause host cell pyroptosis and necrosis and a large pro-inflammatory response. Neutrophils form coalescing foci of inflammation that are unable to combat the massive bacterial growth, and acute bronchopneumonia rapidly overtakes the host. Depletion of neutrophils early following infection increases the sensitivity of mice to pneumonic plague suggesting that neutrophils are, at least initially, active against *Y. pestis* [9].

The type III secretion system (T3SS) is responsible for the injection of anti-host effector proteins, collectively known as Yops, into phagocytic and other mammalian cells and is essential for virulence [10]. Upon host cell contact, insertion of a translocation pore into the plasma membrane allows injection of Yops from the bacteria to the host cytosol [11], [12], [13]. Each Yop likely has a distinct role in the pathogenesis of infection because *Y. pestis* strains lacking individual effector proteins have varied virulence defects ranging from mild to severe [14], [15], [16], [17], [18], [19]. Yop-mediated host cell death and modulation of inflammatory responses have been demonstrated and are important to the pathogenesis of plague. The T3SS effector protein YopJ is a deubiquitinase and acetyltransferase whose activity blocks signaling by mitogen activated protein kinase, prevents NF-κB activation and leads to decreased production of anti-apoptotic proteins and pro-inflammatory cytokines [20], [21], [22], [23]. Yet in spite of these immune-modulating activities, YopJ is largely dispensable for virulence [18]. Moreover, the evolution of *Yersinia* towards increased virulence includes reductive secretion of YopJ suggesting selective pressure against YopJ activity for the development of plague [24], [25].

Translocation of effector Yops is believed to be controlled by YopK, whose activity has been shown to modulate the size of the translocation pore in red blood cells and epithelial cells [26], [27]. YopK is translocated into host cells where it localizes to the plasma membrane [27], [28]. In addition, recent evidence supports a role for YopK in modulating inflammatory responses. Insertion of the translocation pore by a *Y. pseudotuberculosis* strain lacking most of the Yop effectors resulted in activation of caspase-1 and the inflammasome, subsequent production of pro-inflammatory cytokines and pyroptotic cell death [29], [30], [31], [32]. YopK was therefore proposed to remain associated with the translocation pore where it could suppress inflammasome activation [33]. Nevertheless, *in vivo*, YopK was dispensable for caspase-1-mediated host defense suggesting additional function for YopK in *Y. pseudotuberculosis* virulence. *Y. pestis* is believed to evade inflammasome activation at least in part, due to the presence of a non-canonical lipopolysaccharide structure, not present in *Y. pseudotuberculosis*, which does not stimulate Toll-like receptor 4 signaling [34], [35].

In this work, we found that YopK mediates inflammatory responses in a murine model of pneumonic plague. We show that YopK is necessary for YopJ-dependent activation of apoptosis of monocyte-like macrophages but for alveolar macrophages, deletion of YopK resulted in increased apoptosis. Using small molecule inhibitors, we show that YopK activity in RAW 264.7 cells is independent of caspase-9, acting upstream of caspase-8 in the extrinsic apoptosis pathway. *In vivo*, YopK is necessary for virulence, and its deletion caused a 5,000-fold increase in lethal dose. YopK-dependent modulation of macrophage apoptosis was observed during the first 24 hrs of infection and led to recruitment of macrophages, prevention of dendritic cell migration and subsequent progression of inflammation and disease. In contrast, lung inflammation and congestion were observed early but were quickly followed by a resolution program in the absence of YopK. Pro-inflammatory cytokine production was not affected by YopK *in vitro*. Nevertheless, elevated levels of MCP-1 persisted throughout the *yopK* infection *in vivo* and the MCP-1 receptor, CCR2, was involved in host defense. Together, the data suggest that YopK modulates inflammation through the control of macrophage and perhaps other immune cell apoptosis for the rapid development of pneumonic plague.

## Results

### *Y. pestis* KIM D27 *yopK* is required for caspase-3 activation during infection of murine macrophages *in vitro*

**Figure 1:**
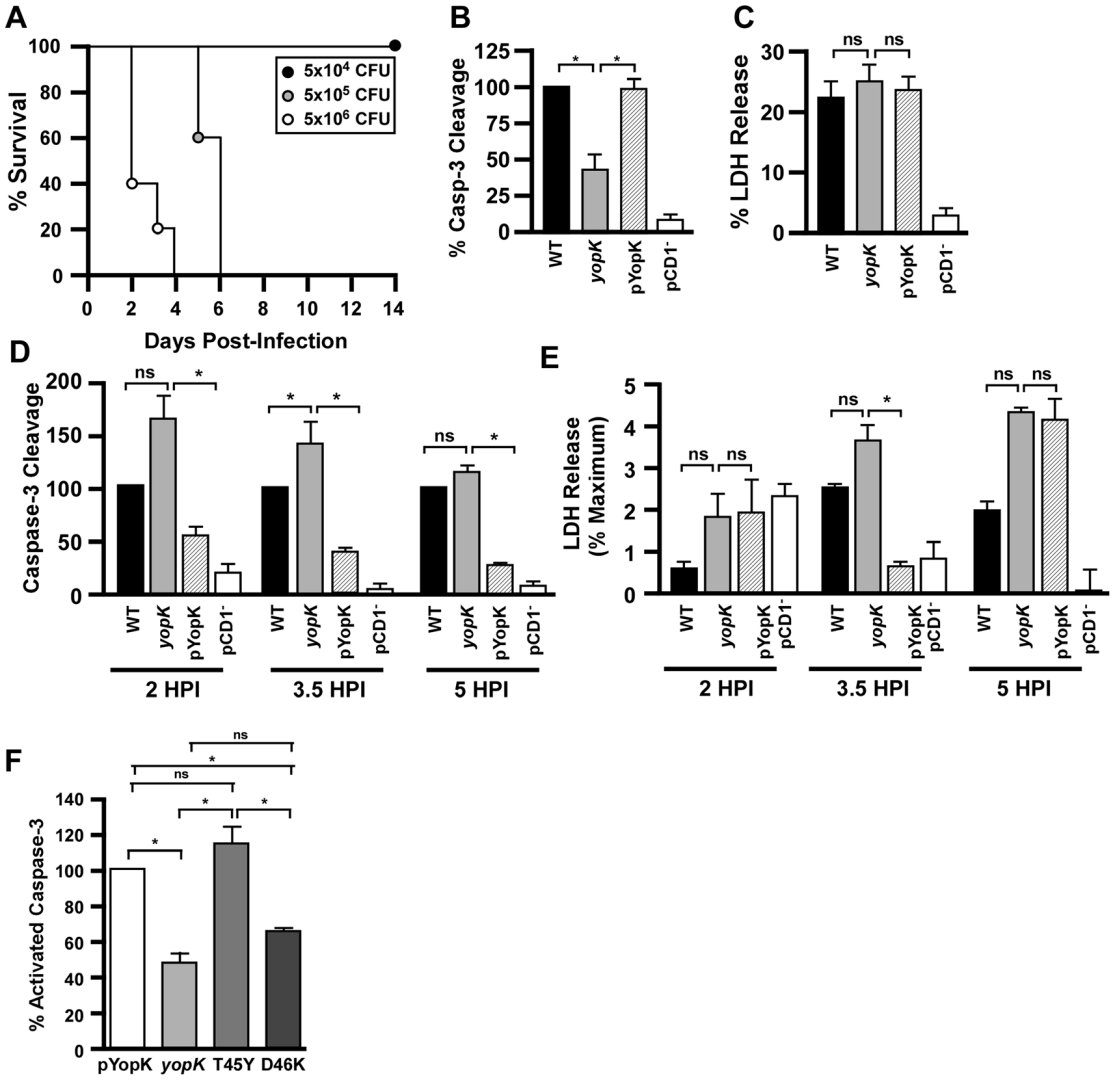
YopK modulates caspase-3 activation *in vitro* in *Yersinia pestis* infected macrophages. A) Intravenous infection of C57BL/6 mice with the indicated doses of KIM D27 *yopK* for determination of 50% lethal dose (data shown are representative of two trials; n = 8–10 mice per group total). RAW 264.7 cells were infected with *Y. pestis* KIM D27, *yopK*, pYopK and CO92 pCD1^−^, (B) harvested after 3.5 hours and assayed for caspase-3 activation (shown are the mean values from four trials, each sample run in duplicate) or (C) harvested after 4 hours for release of lactate dehydrogenase (LDH) (shown are the mean values from nine trials, each sample run in duplicate). (D–E) MH-S cells, an alveolar macrophage cell line, were infected with KIM D27, *yopK*, pYopK, and KIM6- (pCD1^−^) and assayed for caspase-3 activation (D) and LDH release (E) at 2, 3.5, and 5 HPI, shown are the mean values from two (5 HPI), four (2 HPI) or six (3.5 HPI) trials, each sample run in duplicate. (F) RAW 264.7 macrophages were infected at an MOI of 20 for 3.5 hours with *Y. pestis* KIM D27*yopK* complemented with wild type *yopK* or the T45Y or D46K mutants; data shown are the mean values collected in 2–5 independent trials, with each sample run in duplicate. Caspase data are reported as a percentage of caspase activation by WT *Y. pestis* KIM D27; LDH data are reported as a percent of total LDH released following lysis of macrophages that were not infected. Data from each trial were evaluated for statistical significance using one-way ANOVA followed by the Tukey post-hoc test, *p<0.05, ns not significant.

We generated a deletion of the *yopK* open reading frame in *Y. pestis* KIM D27 using homologous recombination and the suicide plasmid pCVD442 [36]. The resulting strain was analyzed by PCR and Western blot, confirming the deletion of *yopK* and the presence of all three virulence plasmids (data not shown). This mutant strain was also found to be highly attenuated in an intravenous model of septicemic plague, with a calculated LD_50_ of 1.5×10^5^ CFU, a 1,500-fold increase over the LD_50_ of the wild type (WT) *Y. pestis* KIM D27 strain (Figure 1A) [14].

Previous data on the function of YopK employed *in vitro* infection models of epithelial cells where increased injection of effector Yops has been demonstrated using cell fractionation and reporter assays examining a subset of Yops [26], [27]. However, it is known that during infection, *Y. pestis* targets phagocytic cells [37]. We therefore sought to characterize the *yopK* mutant *in vitro* using macrophage infection models to better understand the potential impact of YopK in plague. Towards this end, we employed functional assays and first measured the induction of apoptosis in RAW 264.7 cells, a mouse monocyte-macrophage cell line. Caspase-3-dependent apoptosis of macrophages is caused by the T3SS effector YopJ, which has been shown to have increased translocation in the absence of *yopK* [27]. Thus, we anticipated that more caspase-3 activation would be observed in the *yopK* mutant due to the increased effector Yop translocation. Unexpectedly, however, the *yopK* mutant did not cause increased cleavage of caspase-3 but rather significantly less cleavage was observed (Figure 1B). This phenotype could be complemented by expressing YopK on a low copy plasmid from its native promoter. In contrast, *Y. pestis*-induced lysis of RAW 264.7 macrophages was not significantly affected by YopK, and 25–30% LDH release was observed following infection by WT and *yopK* mutant *Y. pestis* (Figure 1C). Host-cell lysis was dependent on the T3SS, however, and only 5% LDH release was observed following infection of RAW macrophages with *Y. pestis* lacking the T3SS virulence plasmid pCD1.

We also tested whether MH-S cells, a murine alveolar macrophage cell line, induced increased amounts of YopK-dependent caspase-3 activity following *Y. pestis* infection. In sharp contrast to RAW 264.7 cells, MH-S cells infected with *Y. pestis yopK* had significantly increased caspase-3 cleavage, consistent with increased translocation of YopJ due to the deregulation of the translocation pore (Figure 1D). When *yopK* expression was complemented on a recombinant plasmid, however, levels of caspase-3 cleavage were significantly decreased to levels below that caused by the WT strain. This result is also consistent with a role for YopK as a regulator of pore size in the plasma membrane of MH-S cells where increased expression and translocation of YopK would be predicted to result in decreased injection of Yops. Furthermore, LDH release by infected MH-S cells was overall low, and only 3–5% LDH release was observed following infection by WT and *yopK* mutant *Y. pestis* (Figure 1E). This low level of MH-S cell lysis was dependent on the T3SS as less than 1% LDH release was observed following infection by pCD1 mutant bacteria.

Previous data indicated that YopK amino acid residue D46 was important for function whereas residue T45 was dispensable [38]. We therefore engineered these point mutations into the yopK complementing plasmid and tested them for the ability to complement the *yopK* mutant in inducing apoptosis of RAW macrophages. Indeed, mutation of D46 resulted in loss of YopK function and no complementation of apoptosis was observed (Figure 1F). In contrast, T45 was dispensable and this mutant fully complemented apoptosis of RAW macrophages. Together, the genetic data support a role for YopK in the induction of apoptosis in RAW macrophages.

### *Y. pestis* induces caspase-3 activation in RAW macrophages through two distinct pathways

**Figure 2:**
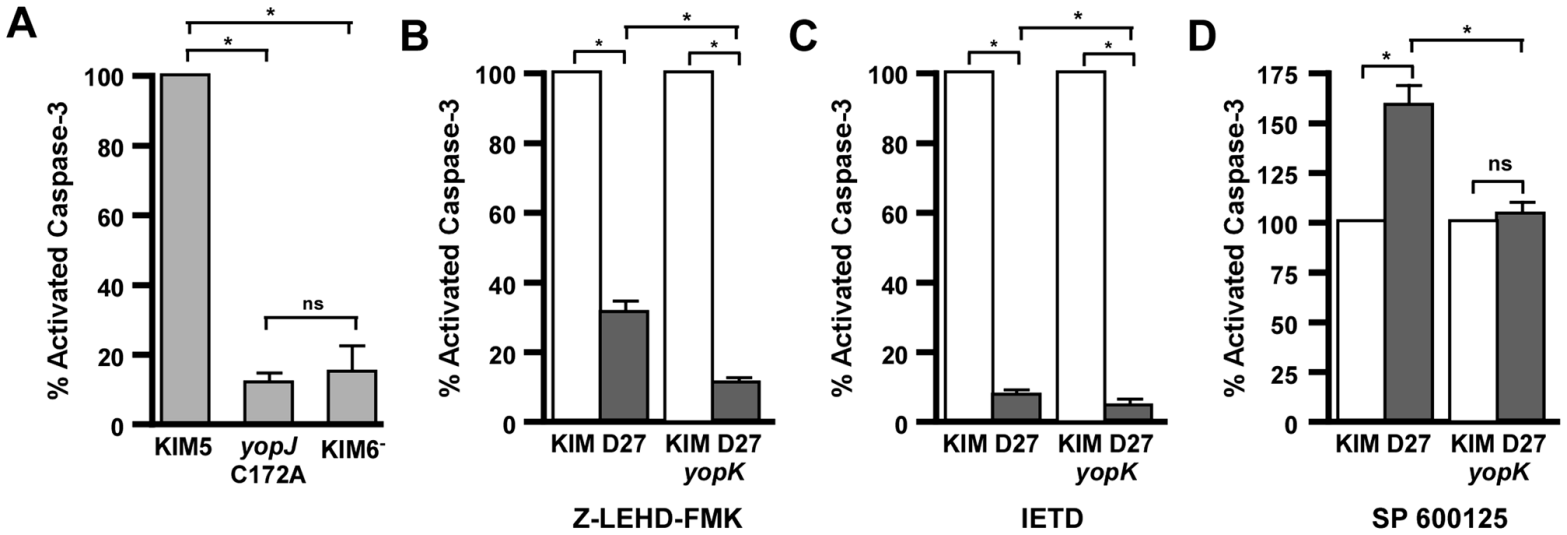
YopK contributes to caspase-3 cleavage through the extrinsic pathway. A) RAW 264.7 macrophages were infected with KIM5, KIM5 *yopJ*C172A, or KIM6^−^ at an MOI of 20 and assayed for caspase-3 activity after 3.5 hours of infection. Data shown are the mean of four biological replicates, collected in two independent trials, and are shown as a percentage of caspase-3 activity from WT-infected cells. B–D) RAW 264.7 macrophages were left untreated (white bars) or treated with inhibitors (grey bars) for (B) caspase-9 (Z-LEHD-FMK), (C) caspase-8 (IETD), or (D) JNK (SP600125) and infected with either *Y. pestis* KIM D27 or KIM D27 *yopK* at an MOI of 20. After 3.5 hours of infection, cell pellets were analyzed for cleaved caspase-3. Data shown are the mean of four replicates, collected in two independent trials and are shown as percent of untreated for each strain. Statistical significance by one-way ANOVA followed by Tukey post-hoc test, *p<0.05, ns not significant.

*Y. pestis yopK* has previously been shown to translocate increased amounts of YopJ in CHO cells, yet in RAW macrophages, the *yopK* mutant induced decreased caspase-3 activation [27]. This result suggests that YopK may be necessary for YopJ activity in specific cells or that YopK has an independent role in inducing apoptosis that might be additive to YopJ. However, previously published data suggests apoptosis of *Y. pestis*-infected RAW macrophages requires YopJ [39]. To confirm that apoptosis of RAW macrophages requires YopJ in our assay, we infected RAW macrophages with *Y. pestis* carrying a point mutation in the catalytic site of YopJ (C172A) which abolishes its ability to induce apoptosis [31]. Consistent with previous reports, YopJ is necessary for apoptosis of macrophages and, upon infection with the *yopJ* mutant strain, no caspase-3 activation was observed (Figure 2A). Therefore, while YopK is necessary for maximal apoptosis in RAW cells, YopJ is absolutely required.

Previously, inhibition of caspase-8 and caspase-9 revealed that *Y. pseudotuberculosis* YopJ induces apoptosis through both the intrinsic and extrinsic pathways [40]. We therefore asked whether YopK was necessary for both of these activities. Caspase-3 cleavage can be activated by caspase-8 whose auto cleavage from pro-caspase-8 occurs following formation of a complex involving the adaptor protein FADD which is normally initiated through binding of Fas or TNF receptors [41]. YopJ is necessary for inducing formation of the DISC in the absence of receptor binding [42]. In lymphocytes and perhaps other immune cells, formation of the FADD-caspase-8 complex, the death-inducing signaling complex (DISC) leads to caspase-3 activation directly, while in most cells, caspase-3 activation requires amplification through caspase-9 and the intrinsic apoptosis pathway [43].

We therefore treated RAW macrophages with inhibitors to caspase-8 and -9 and measured the impact on *Y. pestis*-induced activation of caspase-3. Pre-treatment of macrophages with caspase-9 inhibitor Z-LEHD-FMK partially blocked caspase-3 activation and 30% remained following infection of RAW cells by WT *Y. pestis* (Figure 2B). This suggests that *Yersinia*-induced caspase-3 activation in RAW macrophages is partially dependent on caspase-9. In contrast, the caspase-9 inhibitor had a more severe effect on apoptosis caused by the *yopK* mutant bacteria where caspase-3 activation was reduced to background levels. This suggests that the caspase-3 activity caused by the *yopK* mutant may be due solely to caspase-9-dependent signaling whereas when YopK is present, the extrinsic, caspase-9-independent pathway may be induced. Consistent with this hypothesis, pre-treatment of macrophages with caspase-8 inhibitor (IETD) abolished caspase-3 activity following infection by both WT and *yopK* mutant *Y. pestis* suggesting caspase-8 is absolutely required for caspase-3 activation following *Y. pestis* infection (Figure 2C). Together, the data suggest that YopK acts upstream of caspase-8 to modulate YopJ-dependent apoptosis of RAW macrophages.

Following *Y. pestis* infection, inhibitors of c-jun N-terminal kinases (JNK) increase caspase-3 activation of RAW and J774 macrophages in a manner dependent on the acetylation activity of YopJ [44]. We asked whether this required YopK. SP600125 pre-treatment caused a 50% increase in caspase-3 activation of RAW macrophages following WT infection, similar to previously published data (Figure 2E). In contrast, SP600125 had no effect on infection by the *yopK* mutant as no detectable differences were observed between treated and untreated macrophages. Thus, like YopJ, YopK is required for the JNK inhibitor to induce apoptosis in infected RAW macrophages. Taken together the inhibitor data suggest that *Y. pestis* induces YopJ-dependent apoptosis of RAW macrophages through the intrinsic and extrinsic pathways, with a requirement for YopK in the extrinsic pathway.

### *Y. pestis* CO92 *yopK* is severely attenuated during pneumonic plague

Because macrophages are key mediators of the innate and adaptive immune systems, and their apoptosis is likely to have profound effects on these systems *in vivo*, we were interested in understanding how the contribution of YopK to apoptosis affected inflammatory responses during pulmonary infection. Since the *Y. pestis* KIM D27 parent strain is a non-pigmented mutant, it is attenuated and cannot cause pulmonary disease [45]. In order to better understand the role of YopK in pathogenesis, we studied the fully virulent *Y. pestis* CO92 strain which is capable of causing primary pneumonic plague. Towards this end, we generated a *yopK* null mutation in *Y. pestis* CO92 using the strategy described above. This strain was characterized by PCR, confirming the absence of *yopK* and presence of pCD1, pPCP and pMT1 (data not shown). We measured the LD_50_ of this strain following intranasal infection of BALB/c mice as 1.5×10^6^ CFU, a 5,000-fold increase in lethal dose over that of the parent CO92 strain (Supplemental Figure S1A). This increase appears more severe than the LD_50_ measured in the septicemic plague model and, together with previously published data, suggests that YopK has an essential function for all forms of plague [14]. Importantly, the LD_50_ for both CO92 and KIM D27 *yopK* mutant strains is less than that observed following pulmonary infection of *Y. pestis* lacking the type III secretion system (pCD1^−^), a strain that cannot cause disease in mice indicating the virulence defects observed in our strains are not due to second site mutations that result in loss of T3SS activity.

Following infection with lethal challenge doses of *yopK*, some mice succumbed to disease in 3 to 4 days post-infection, a similar time course as wild type CO92 at 100-fold lower challenge dose, while about 10% died later (5 to 14 days post-infection). The mice that succumbed to disease early appeared to be suffering from primary pneumonic plague, defined as multi-focal bacterial colonies in the lungs and neutrophil congestion in the parenchyma (Supplemental Figure S1B–C). Those mice that succumbed to disease later appeared to have developed and succumbed to septicemic plague, with fewer bacteria in the lungs, interstitial pneumonia, and massive liver and spleen necrosis and inflammation (Supplemental Figure S1D–F). Bacterial titers and severity scores for lungs, liver and spleen of moribund mice were determined (Supplemental Table S3). These mice had in common little to no damage to or colonization of the spleen, but an overall systemic infection.

### Clearance and resolution of inflammation following infection of *Y. pestis* yopK

**Figure 3:**
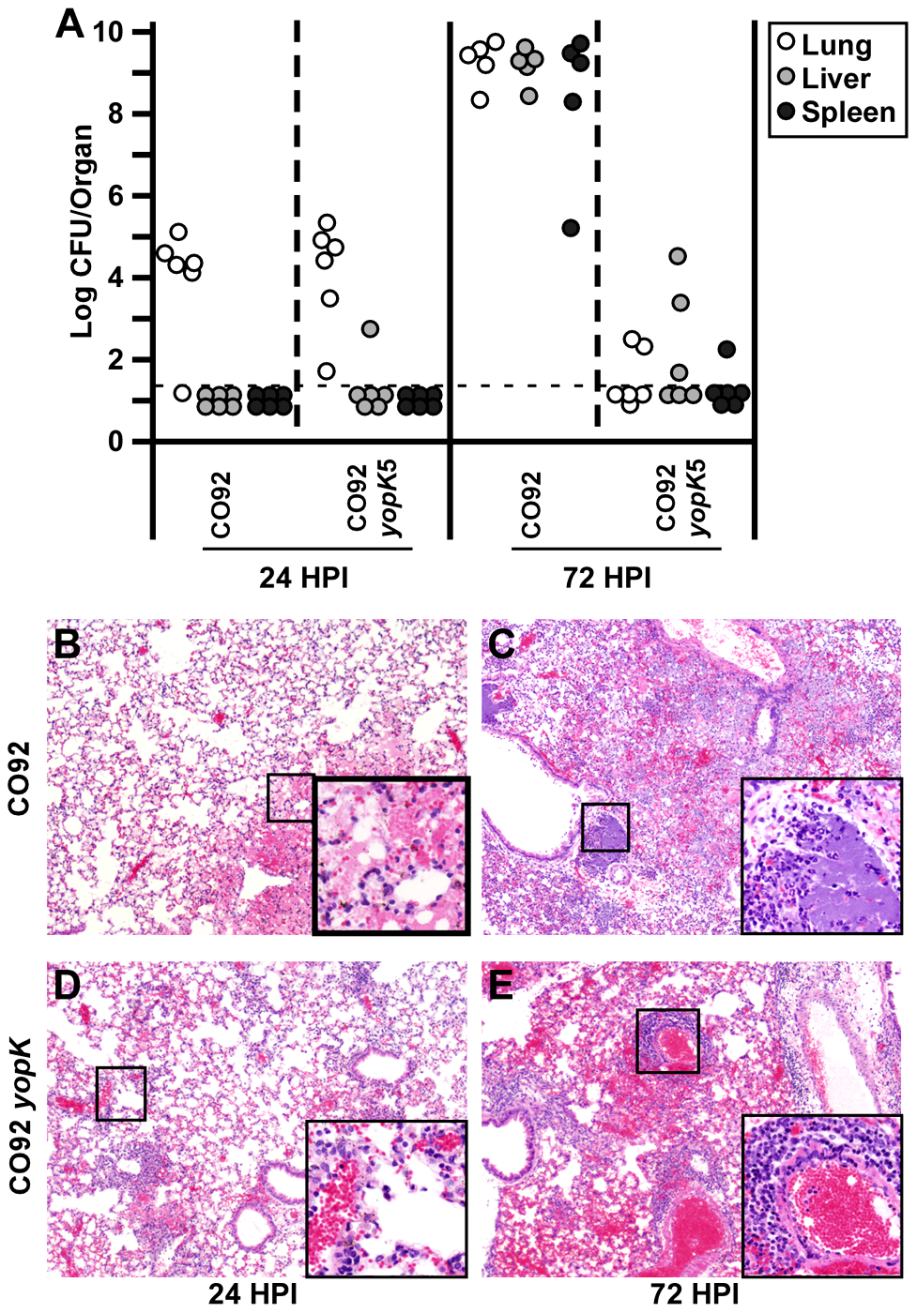
Similar early inflammation following pulmonary challenge with WT and *yopK Y. pestis* is resolved in the absence of YopK. BALB/c mice were challenged by intranasal infection with 1×10^4^ CFU of WT *Y. pestis* CO92 or 1×10^6^ CFU of CO92 *yopK*. At 6, 24 and 72 HPI, mice were euthanized and lungs, livers, and spleens were divided with one-half tissue analyzed for bacterial titers (A) including lungs (open), liver (grey) and spleen (black) and the second half formalin fixed, sectioned and stained for histochemistry (B–E). H&E stains of WT-infected lungs (B–C) and *yopK*-infected lungs (D–E) from 24 (B,D) and 72 (C,E) HPI. Data shown were collected in two independent experiments, n = 6 mice per time point per strain. Statistical significance of differences in bacterial titer of WT- and *yopK*- infected lungs was analyzed by unpaired Student's t-test, *p<0.05. (B–E) Boxes show 4× magnified section of indicated area.

To identify host responses that lead to clearance of *yopK* mutant *Y. pestis*, we next performed a 72 hour time course experiment. Due to the large attenuation of the *yopK* mutant, comparisons with wild type are subject to misinterpretation because of the growing differences in clinical state and bacterial load in the tissues over time. We therefore studied two challenge doses: one equivalent to 0.67 LD_50_ for *yopK* (1×10^6^ CFU) and the second equivalent 30 LD_50_ for wild type (1×10^4^ CFU, a dose which causes no symptoms when used for the *yopK* mutant). This allowed us to assess similar clinical states of infection caused by both strains of bacteria. At 6 hours post-infection (HPI), increased bacterial load for *yopK* was observed compared to WT due to the 100× higher inoculating dose (data not shown). At 24 HPI, however, both strains colonized the lungs at similar levels (Figure 3A). Strikingly, at this time point, pathology in the lungs of both wild type and *yopK* infected mice were similar with mild to moderate infiltration of neutrophils and little tissue damage (Figure 3B, D). In contrast, at 72 HPI, the divergence of the two strains was notable, with wild-type bacteria replicating to high titer while *yopK* mutant bacteria were undergoing clearance. Congestion in *yopK*-infected lungs at 72 HPI was mild, and either interstitial or consisted of lymphocytes around blood vessels near the airways, while in WT-infected lungs, inflammation was more severe and predominantly neutrophils were present (Figure 3C,E). Wild type-infected mice also had visible bacterial colonies in the hepatic sinusoids of the liver as well as the red pulp of the spleen while the *yopK* mice had smaller inflammatory foci in the liver and spleen (Supplemental Figure S2A–D). Together, the pathology of the *yopK* infection suggests that these bacteria may cause an alteration of the host inflammatory response compared to that caused by wild type *Y. pestis*.

In order to identify the cellular infiltrate in the lungs during the later stage of infection, we analyzed these tissue sections by immunohistochemistry. Formalin fixed lung tissues from 72 HPI were stained with NIMP R14 (a monoclonal antibody that recognizes Ly6G/6C on the surface of monocytes and neutrophils), F4/80, CD3 or B220 and analyzed by microscopy. Wild type bacteria induced an inflammatory response in the lungs that appeared primarily composed of Ly6G/6C^+^ cells (Supplemental Figure S3) suggesting active neutrophil recruitment, while in the liver and spleen, fewer neutrophils were observed (Supplemental Figure S4). In contrast, fewer Ly6G/6C^+^ staining cells were present in *yopK* infected mice, and instead, F4/80^+^, CD3^+^ and B220^+^ stained cells were found especially in the lungs indicating recruitment of macrophages, T cells and B cells. Together, the results suggest a dramatic difference in the inflammatory response between WT- and *yopK*-infected mice at 72 HPI. Correlation between divergence in inflammatory cell recruitment and reduction in bacterial load suggests that the primary role of YopK in virulence may be immunomodulatory.

### YopK is associated with early macrophage apoptosis and modulation of the innate immune response *in vivo*

**Figure 4:**
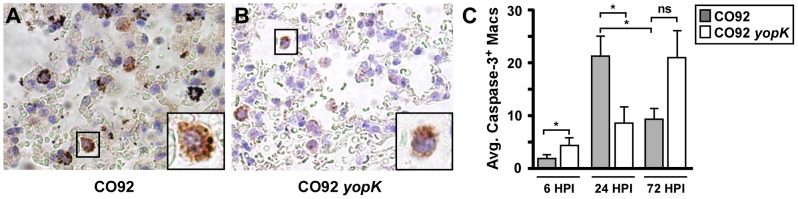
Increased apoptosis of lung macrophages is associated with clearance of *Y. pestis yopK*. Tissue sections from 6, 24, and 72 HPI with 1×10^4^ CFU of WT (A) or 1×10^6^ CFU of *yopK* (B) were stained by immunohistochemistry with antibodies to cleaved caspase-3. Apoptotic macrophages (A–B) were the most prominent positively staining cells; images are representative and shown at 40× magnification with 100× inset. C) Apoptotic macrophages were counted in 10 non-overlapping fields on blinded slides at 40× magnification. Data shown are the mean number of caspase-3-positive macrophages per field of each mouse, averaged over 6 mice per group *p<0.05, ns not significant, evaluated by unpaired Student's t-test. Data were collected from two independent experiments.

To determine if YopK-dependent caspase-3 activation occurs *in vivo*, we also stained lung tissue sections from 6, 24 and 72 HPI with labeled antibodies to cleaved caspase-3 and analyzed them by microscopy. Initial assessment revealed relatively frequent caspase-3 positive macrophages in both WT- and *yopK*-infected lungs (Figure 4A–B). We counted caspase-3 positive macrophages in 10 non-overlapping fields of lungs at 40× magnification. At 6 HPI, a modest, but significant increase in caspase-3-positive staining was observed following *yopK* infection compared to WT suggesting an increase in apoptotic cells (Figure 4C). This observation is similar to that observed in MH-S cells, however it is important to note that at this time point, there are increased numbers of *yopK* bacteria compared to WT which may be responsible for this observation. In contrast, at 24 HPI when bacterial titers of WT and *yopK* were highly similar, lungs harvested from mice infected by WT contained greater than 2-fold more macrophages undergoing apoptosis compared to *yopK* infected mice. This suggests that YopK may induce increased apoptosis of macrophages *in vivo*, correlating with the progressing development of pneumonia in WT-infected mice. In contrast, at 72 HPI, mice infected by WT bacteria (now progressing towards lethality), displayed significantly reduced caspase-3 positive macrophages compared to 24 HPI. At this time point, the *yopK*-infected mice have begun to resolve inflammation and likely harbor many fewer bacteria than the WT-infected mice. Overall, these results are consistent with a role for YopK in modulating macrophage apoptosis early following infection, as seen *in vitro*.

**Figure 5:**
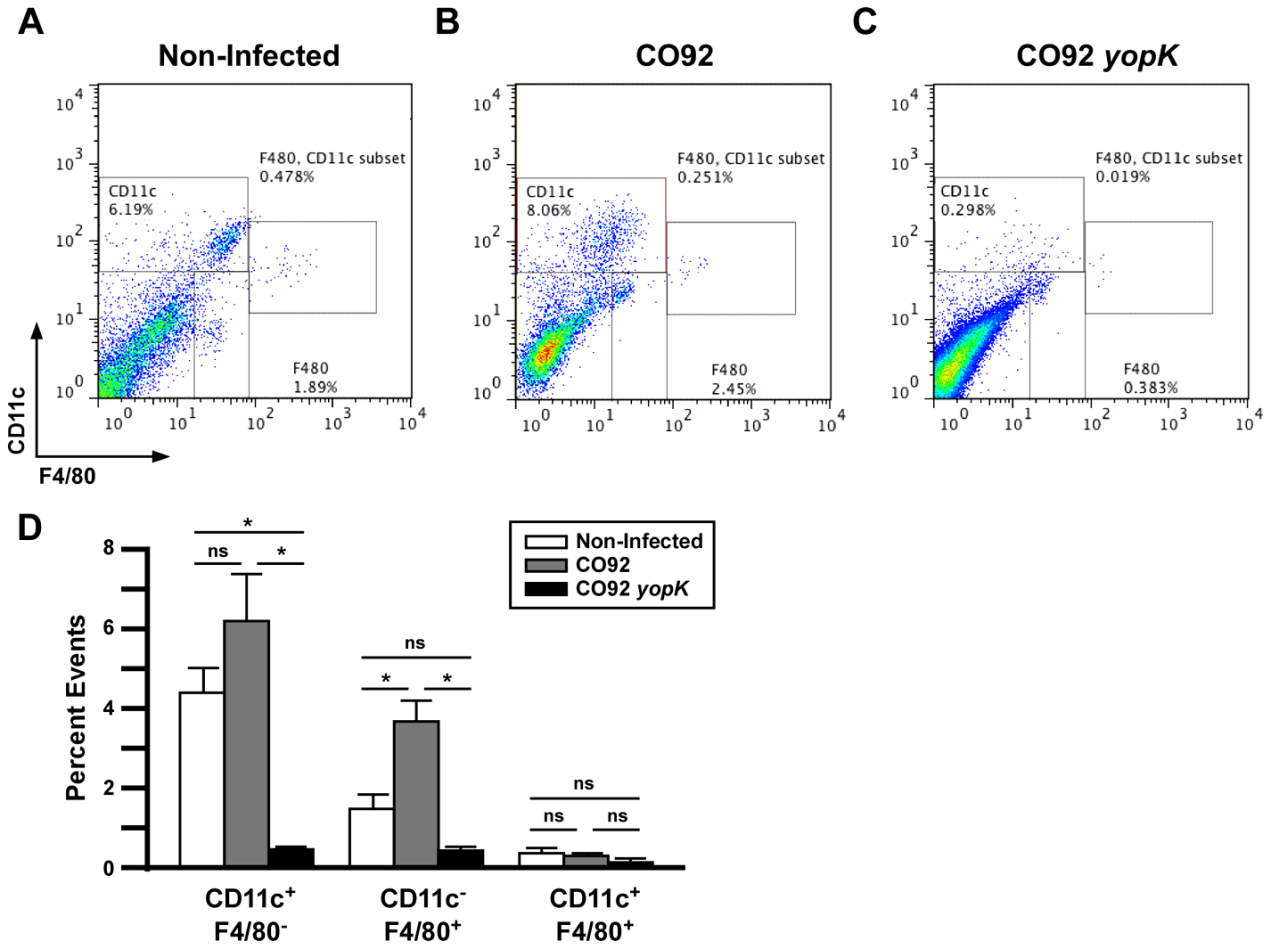
The presence of YopK leads to increased populations of dendritic cells and macrophages in the lungs. BALB/c mice were challenged by intranasal infection with 1×10^4^ CFU *Y. pestis* CO92 or 6×10^5^ CFU CO92 *yopK.* At 24 HPI, infected lungs were isolated and homogenized for analysis by flow cytometry. A–B) Gating of events based on CD11c-PE and F4/80-FITC in naive (A), CO92- (B), and *yopK*- (C) infected lungs. D) Graphical representation of average numbers of CD11c^+^F4/80^−^, CD11c^−^F4/80^+^, and CD11c^+^F4/80^+^ cells in the lungs of CO92- or CO92 *yopK*-infected mice, *p<0.05, evaluated by one-way ANOVA followed by Tukey post-hoc test. n = 3–4 mice per group.

We quantified the number of lung and alveolar macrophages and dendritic cells by flow cytometry. Lung homogenates of BALB/c mice were prepared at 24 HPI and stained with CD11c and F4/80 (Figure 5A–C). Alveolar macrophages (CD11c^+^F4/80^+^) appeared similar in number between naïve mice and WT- or *yopK*-infected mice (Figure 5D). Strikingly, lung macrophages (CD11c^−^F4/80^+^) were increased in WT- but not *yopK*-infected mice while dendritic cells (CD11c^+^F4/80^−^) were decreased in *yopK*-infected mice. Together, the data suggests that YopK activity *in vivo* leads to recruitment of macrophages, resulting in progressing bronchopneumonia, while the absence of *yopK* caused an alternative inflammatory program that correlated with bacterial clearance and no disease.

### YopK is necessary for controlling CCR2-dependent inflammatory responses during the early stage of infection

**Figure 6:**
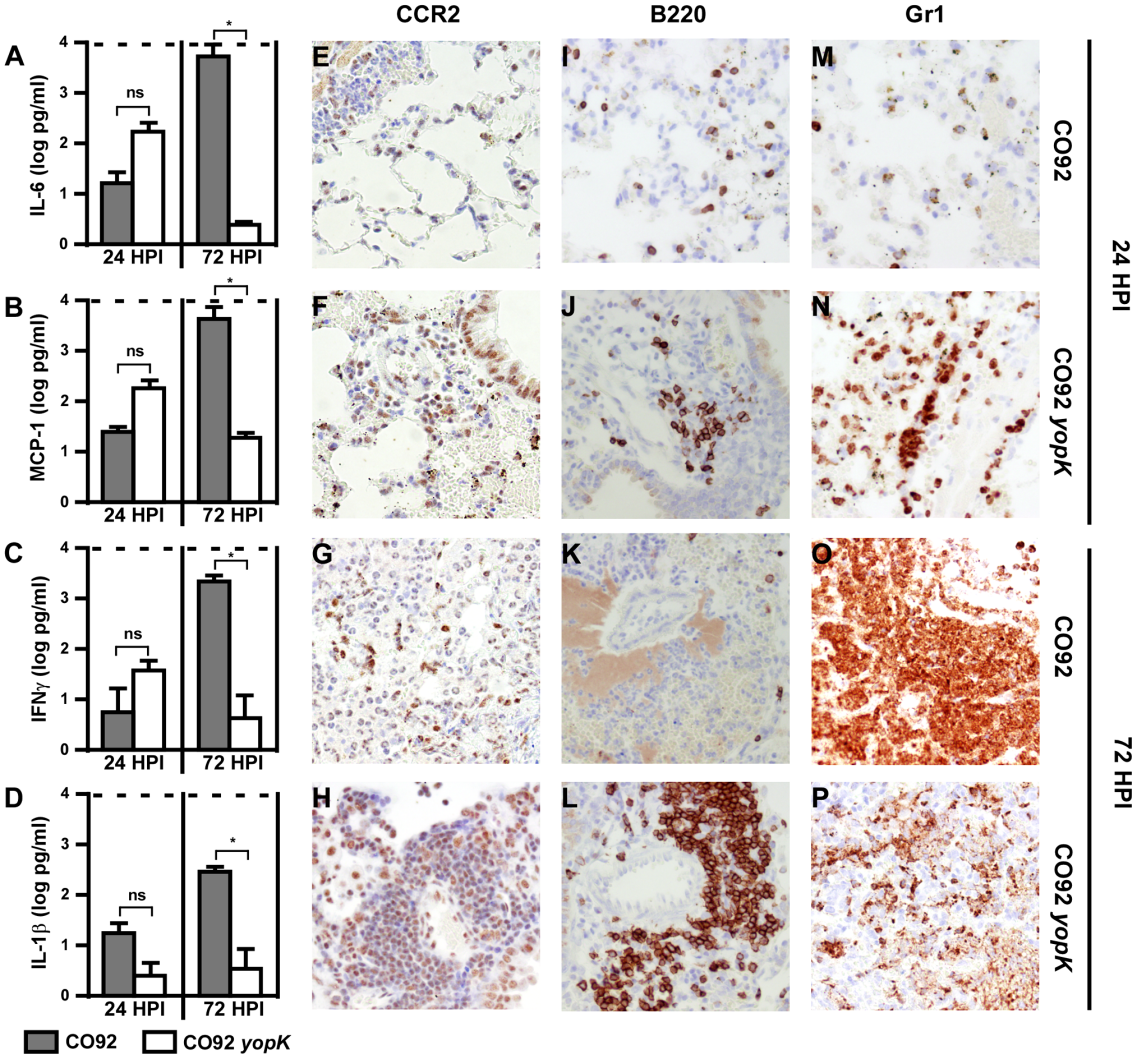
Early induction of pro-inflammatory cytokines converts to a resolution response as the *yopK* infection clears. A–D) BALB/c mice were challenged by intranasal infection with 1×10^4^ CFU of WT (grey bars) or 1×10^6^ CFU *yopK* (white bars) and serum cytokine levels were determined at 24 and 72 HPI by 13-plex cytokine array. (A) IL-6, (B) MCP-1, (C) IFNγ, and (D) IL-1β; *p<0.1, evaluated by unpaired Student's *t*-test. Tissues from these mice were analyzed by immunohistochemistry by staining with CCR2 antibodies (E–H), CD45R/B220 (I–L), and Gr1 (M–P); WT CO92 at 24 HPI (E,I,M) and 72 HPI (G,K,O), and *yopK* at 24 HPI (F,J,N) and 72 HPI (H,L,P). All images are representative and were taken at 40× magnification. Data were collected in two independent experiments, n = 6 mice per group.

YopK-induced apoptosis of macrophages would be expected to impact bacterial clearance directly, contribute to neutrophil-induced tissue injury and/or modulate inflammatory responses [46], [47]. To distinguish between these possibilities, we sought to determine the overall impact of YopK on immunomodulation during infection. Towards this goal, we analyzed serum cytokines from mice infected with WT and the *yopK* mutant for a panel of 13 cytokines and chemokines. When comparing WT- and *yopK*-infected mice at 24 HPI, we found similar, low levels of pro-inflammatory cytokines in the serum (Figure 6A–D, Supplemental Table S5). As the infection progressed, WT-infected mice expressed significantly increased amounts of pro-inflammatory and anti-inflammatory cytokines and chemokines compared to the *yopK*-infected mice. Together these data suggest that YopK modulates inflammatory responses but may not affect secretion of inflammatory cytokines or chemokines. Notably, however, *yopK*-infected mice continued to express moderate levels of MCP-1 when most other pro-inflammatory cytokines were no longer detectable.

We therefore stained the formalin-fixed lung sections with antibody to CCR2, the receptor for MCP-1, present on many leukocytes, to determine if CCR2^+^ cells are recruited to the lungs as the *yopK* infection is being cleared. While the *yopK*-infected lungs stained positive for CCR2 as early as 24 HPI, the WT-infected lungs harbored very little CCR2^+^ staining (Figure 6E–F). Likewise, at 72 HPI there were increased numbers of CCR2^+^ cells in the perivascular regions of the *yopK*-infected lungs, while WT-infected lungs continued to harbor little to no CCR2^+^ staining cells (Figure 6G–H). Importantly, the observed changes in the pro-inflammatory cytokine response and inflammatory cell recruitment occurred after the onset of increased macrophage apoptosis, consistent with a model whereby YopK-mediated depletion of macrophages influences inflammation. Furthermore, we also observed increased B220^+^ cells in the perivascular regions of *yopK*-infected lungs at 24 and 72 HPI as compared to WT (Figure 6I–L). In contrast, although Gr1^+^ cells were present in mice infected with either WT or *yopK* mutant *Y. pestis* at 24 HPI, by 72 HPI, Gr1^+^ cells predominated in WT-infected tissues, with *yopK*-infected mice having mainly Gr1^+^ cell debris (Figure 6M–P). Together the results suggest that during pulmonary infection by *Y. pestis yopK*, recruitment of CCR2^+^ cells correlates with bacterial clearance and resolution of inflammation.

### CCR2 is necessary for clearance of *Y. pestis yopK*

**Figure 7:**
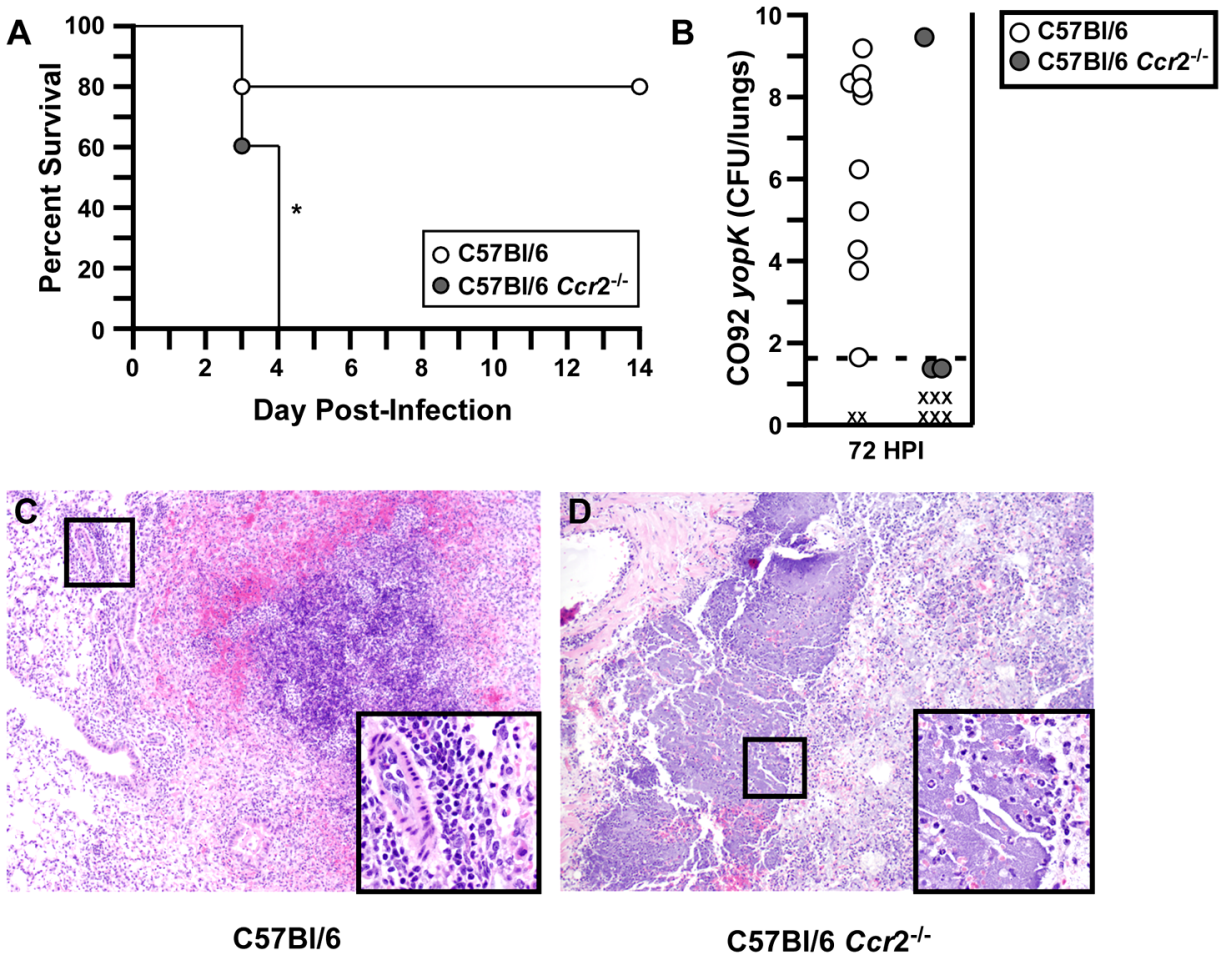
CCR2 contributes to host defense from primary pneumonic plague following intranasal challenge with *Y. pestis* CO92 *yopK*. A) WT C57BL/6 and *Ccr*2^−/−^ mice were challenged by intranasal infection with 5×10^6^ CFU of *Y. pestis* CO92 *yopK* and monitored for development of disease for 14 days. Data shown are representative; n = 10 mice per group, data were collected in two independent experiments. *p<0.05, evaluated by Log rank test. B) Bacterial titers collected from lungs of C57BL/6 (white circles) and C57BL/6 *Ccr*2^−/−^ mice (grey circles) infected intranasally with 5×10^6^ CFU CO92 *yopK* at 72 hpi, data shown from two independent experiments, n = 9–12 mice per group. (C–D) H&E stains of formalin fixed lungs from mice challenged by intranasal infection with 5×10^6^ CFU of *Y. pestis* CO92 *yopK* at 72HPI: (C) C57BL/6, (D) C57BL/6 *Ccr2^−/−^*. Images are representative; boxes show 4× magnified section of indicated area. Data were collected in two independent experiments (n = 9–12 mice per group).

We recently showed that C57BL/6 *Ccr2^−/−^* mice develop pneumonic plague with similar kinetics as wild type mice suggesting that CCR2^+^ cells may play only a minor role during WT infection [48]. However, CCR2 has been shown to be important to host defense against *Y. pestis* mutants lacking YopM [49]. To determine the relevance of CCR2 signaling to host defense against *Y. pestis yopK*, we tested C57BL/6 mice lacking *Ccr2* for sensitivity to pulmonary infection. Groups of 4–6 wild type C57BL/6 were used to determine the lethal dose for C57BL/6 mice, since this is the parent strain background of our *Ccr2^−/−^* mutant mice. These experiments led to a measured LD_50_ of 4.3×10^6^ CFU of *Y. pestis yopK* following intranasal infection, which is approximately 3-fold higher than that measured for BALB/c suggesting C57BL/6 mice may be less susceptible to *Y. pestis yopK* in this model (data not shown). We challenged WT and *Ccr2^−/−^* mice with 5×10^6^ CFU by intranasal infection of *Y. pestis yopK* and survival was monitored for up to 14 days. At this challenge dose, 80% of the WT mice survived the infection while *Ccr2^−/−^* mice were significantly more sensitive (Figure 7A). Knock-out mice that succumbed to infection did so within 4 days, a time course typical of primary pneumonic plague. We examined bacterial load and histopathology in the lungs of WT and *Ccr2^−/−^* mice following intranasal infection with 5×10^6^ CFU *Y. pestis yopK* at 72 HPI. Only three of nine *Ccr2^−/−^* mice survived to that time point, two of which had cleared the infection and the third mouse harbored very high bacterial titer in the lungs, while for WT, more mice survived to 72 HPI, with the surviving mice exhibiting a wide range of titers (Figure 7B). Lungs from moribund *Ccr2^−/.^* mice contained neutrophils and large bacterial colonies in the alveoli suggestive of primary bronchopneumonia while those of wild type mice had smaller bacterial colonies and less inflammation (Figure 7C–D). Together the data suggest that CCR2 signaling helps to limit congestion in the lungs infected by *Y. pestis* lacking YopK.

### YopK does not affect secretion of MCP-1 or IL-1β from macrophages *in vitro*

**Figure 8:**
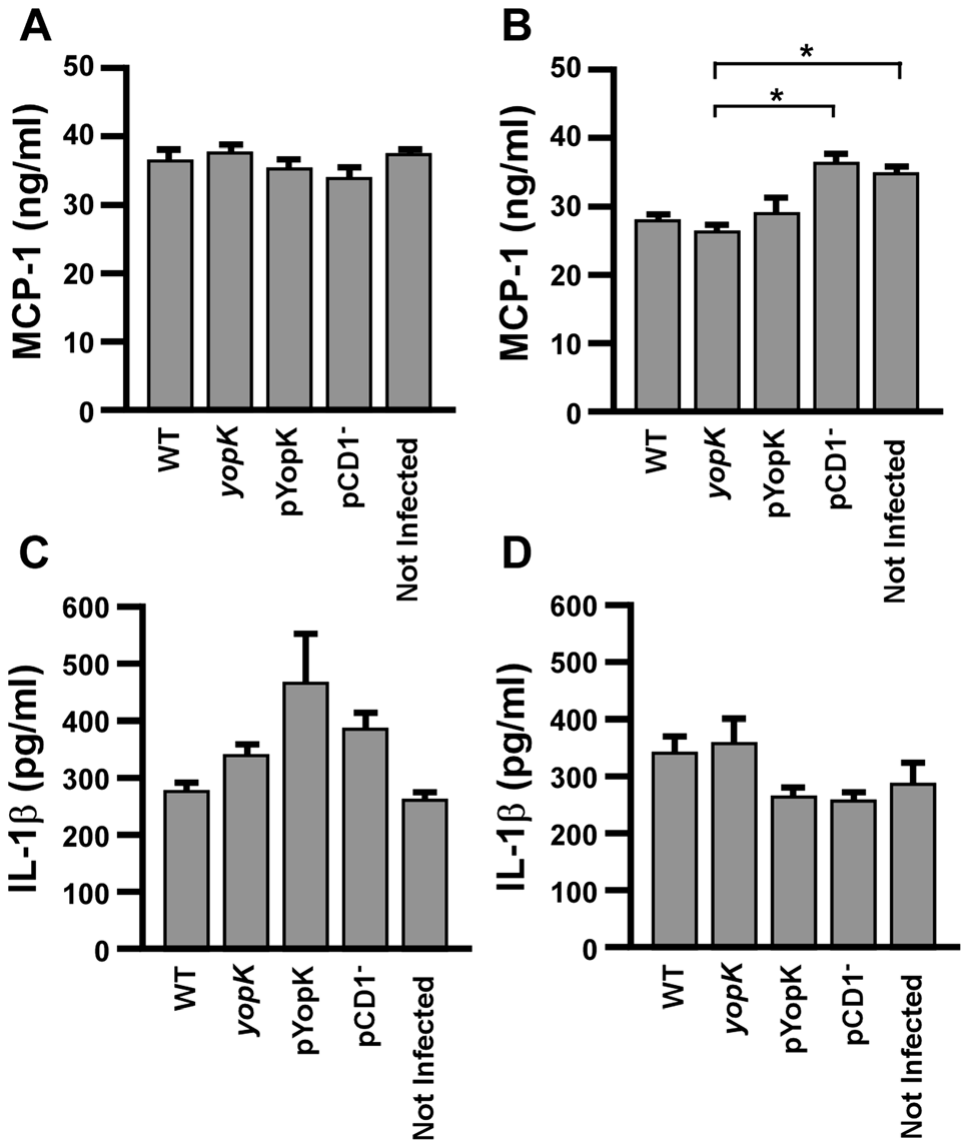
YopK does not directly impact secretion of pro-inflammatory cytokines from macrophages. RAW 264.7 macrophages (A, C) and MH-S alveolar macrophages (B, D) were plated at a concentration of 1×10^6^ cells per well. Cells were activated with IFN-γ 4 hours prior to infection by the indicated strains of *Y. pestis* KIM D27 or CO92 pCD1^−^ at a multiplicity of infection of 20, or were left not infected. After 8 hours of infection, supernatants were analyzed for MCP-1 (A–B) and IL-1β (C–D) by ELISA. *p<0.05, analyzed by one-way ANOVA followed by Tukey post-hoc test. Data shown are representative and collected from two independent experiments with duplicate wells per sample.

To further confirm that the effect of YopK *in vivo* was not due to inhibition of the inflammasome or secretion of pro-inflammatory cytokines, we measured production of MCP-1 and IL-1β following infection of macrophages *in vitro*. Since RAW and MH-S cell lines showed different cell death phenotypes when infected by the *yopK* mutant, we tested each for secretion of MCP-1 and IL-1β at 8 HPI following pre-treatment of macrophages with IFN-γ. No detectable differences in MCP-1 secretion were found between RAW or MH-S cells infected with T3SS-competent strains containing or lacking YopK (Figure 8A–B). Levels of IL-1β produced by RAW and MH-S macrophages were independent of infection and no detectable differences in IL-1β secretion were observed between infected and not infected cells (Figure 8C–D). Together with the data presented in figure 1B and 1E, *Y* *. pestis* YopK does not appear to prevent or cause the induction of the host inflammasome. Overall, the data support a model whereby the function of YopK *in vivo* may be to regulate early apoptosis of immune cells which subsequently reprograms the inflammatory response.

## Discussion

**Figure 9:**
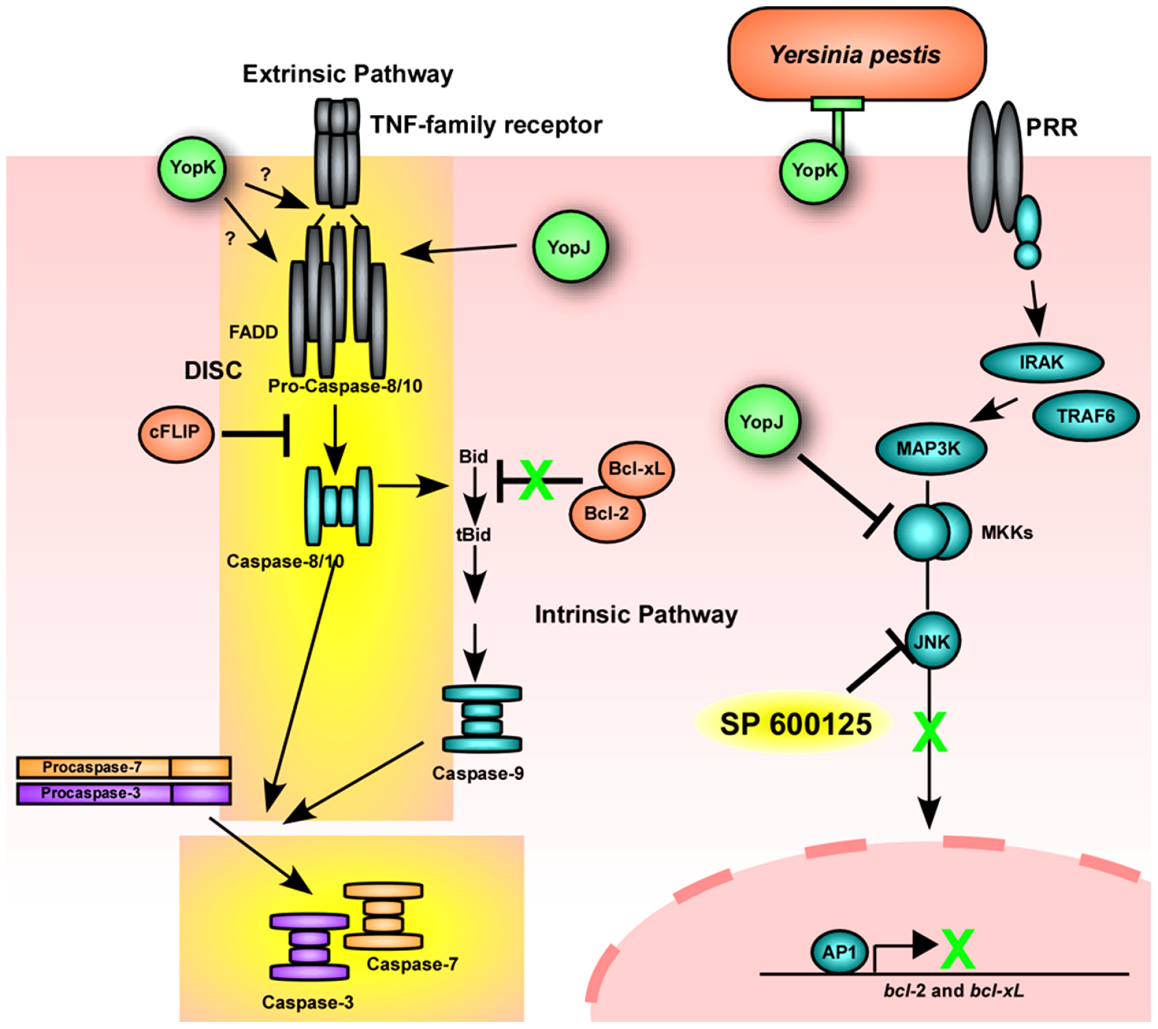
A model for *Yersinia pestis* induced apoptosis of macrophages. Infection of RAW macrophages induces caspase-3-apoptosis through the extrinsic pathway by manipulating host signaling through type III secretion system effectors YopJ and YopK. YopK is located at the plasma membrane where it may control injection of effector Yops but may also be required to induce formation of the DISC death receptor complex, composed of pro-caspase-8 and -10, FADD and cFLIP, which is necessary for activation of caspase-8. In the absence of DISC complex formation, small amounts of active caspase-8 can cleave Bid to tBid in the absence of anti-apoptotic factors whose expression is repressed through the action of YopJ (green X). Formation of the DISC complex is regulated by YopJ. The small molecule SP600125 inhibits JNK phosphorylation, thereby causing reduced production of anti-apoptotic genes. Yellow background denotes the signaling pathway that may be specific to RAW cells and perhaps other immune cells.

The TNF receptor super family is a group of cell surface proteins including TNF-receptor and Fas that are involved in inducing programmed cell death to an extracellular ligand, the extrinsic apoptosis pathway [50]. *Yersinia* infection has been shown to induce the assembly of the pro-caspase-8-containing death receptor complex, known as the DISC, independent of TNF or Fas receptor signaling, thereby activating caspase-8 (Figure 9) [42]. YopJ is essential for activation of apoptosis due to its ability to shut down production of anti-apoptotic proteins and to stimulate assembly of an atypical DISC. YopK is located at the plasma membrane, associated with the translocation pore, where it could regulate, either directly or indirectly, YopJ activity in promoting DISC assembly or downstream signaling.

Resident and recruited macrophages in the lung are believed to be different in their response to apoptotic stimuli, with monocyte-like, recruited macrophages highly sensitive to extrinsic apoptosis while resident alveolar macrophages are somewhat resistant to apoptosis due in part to the expression of NF-κB-induced pro-survival factors [51]. Two populations of macrophages are resident in the mammalian lungs: interstitial and alveolar. Interstitial lung macrophages more closely resemble monocytes, with a greater capacity for immune regulation than alveolar macrophages, and, due to their location in direct contact with epithelial and endothelial cells, they can have pathological impact as well [52]. In contrast, alveolar macrophages exhibit greater functional activity such as increased cytotoxicity, phagocytosis and reactive oxygen species production. MH-S is an alveolar macrophage cell line whereas RAW 264.7 phenotypically more closely resembles monocytes, thus it is conceivable that the differences we observed *in vitro* may have important consequences *in vivo* [52], [53].

Although the *yopK* mutant causes a rapid change to the inflammatory response *in vivo*, we did not identify YopK-dependent pro-inflammatory cytokines produced by infected macrophages *in vitro*. This is especially relevant concerning the proposed function of YopK as a regulator of translocation of effector Yops, a model that predicts increased injection of at least 3 immunomodulators: YopJ, YopH and YopM by *yopK* mutant *Yersinia*. Although YopJ has well-characterized activity in the prevention of NF-κB activation, suppression of TNF-α secretion and induction of apoptosis *in vitro* and *in vivo*, *yopJ* mutant *Y. pestis* exhibited little to no virulence defects in rodent models of plague. Our data suggests that YopK may be important as a regulator of YopJ-mediated apoptosis, and that the dysregulation of apoptosis that occurs in the absence of YopK leads to changes in inflammatory responses *in vivo* that affect the establishment of infection. YopH has been shown to suppress MCP-1 and other pro-inflammatory cytokines *in vitro* [54]. Yet, we did not find decreased MCP-1 secretion between WT and *yopK Y. pestis* infection of RAW or MH-S cells, both of which produced MCP-1 in the absence of infection. Together, the data are accumulating that support a critical role for YopK in modulating inflammatory responses through the control of immune cell death.

CCR2^+^ cells are known to be broadly important to host defense against bacterial infections [55]. CCR2 is expressed by many inflammatory cells and allows for their recruitment to the lungs following infection by many respiratory pathogens. Further, pulmonary infection by *Y. pestis* results in robust MCP-1 secretion (the chemokine for CCR2) in the lungs [56]. Thus, successful respiratory infection and pneumonia that occurs during *Y. pestis* infection requires evasion of early CCR2 responses. Our data and others indicate the T3SS is critical for CCR2 evasion, with three effectors, YopH, YopM, and now YopK implicated in modulating recruitment of CCR2^+^ cells. YopH was shown to suppress pro-inflammatory responses in macrophages, and may delay CCR2-signaled recruitment of immune cells [19]. Recently, YopM was also shown to suppress CCR2 inflammatory responses that would otherwise contribute to bacterial clearance from the liver [49]. Thus CCR2 is a critical mediator of host defense against *Y. pestis* whose effect is blocked by the T3SS.

Many immune cells express CCR2, albeit to varying levels, on the cell surface including monocytes, macrophages, dendritic cells, natural killer T cells, and some B cells. Our observations of the *yopK* infection suggest that all of these cells may be recruited through MCP-1 signaling and one or more of its receptors. CCR2/MCP-1 have been implicated in both bacterial clearance and resolution of inflammation. Although a role for macrophages and monocytes in resolution of inflammation via phagocytosis of apoptotic neutrophils following bacterial clearance is well known, a role for B cells in resolution has not yet been elucidated. The *yopK* pulmonary infection model may therefore provide a novel system for studying the role of B cells and other lymphocytes in the resolution of inflammation.

## Materials and Methods

### Bacterial strains and plasmids

Strains and plasmids used in this study are listed in Supplemental Tables S4 and S5, respectively. All *Yersinia pestis* CO92 strains used were routinely grown fresh from frozen stocks and streaked for isolation onto heart infusion agar (HIA) plates containing 0.005% Congo Red and 0.2% galactose to identify bacteria that retained the pigmentation locus [57], [58]. For pneumonic plague challenge, a single red pigmented colony was used to inoculate heart infusion broth (HIB) containing 2.5 mM CaCl_2_ and grown 18–24 hrs at 37°C, 120 rpm. All handling of samples containing live *Y. pestis* CO92 was performed in a select agent authorized biosafety level 3 laboratory under protocols approved by the University of Missouri Institutional Biosafety Committee. Non-pigmented *Y. pestis* strains were routinely grown fresh from frozen stocks on HIA, followed by aerobic growth at 28°C in HIB overnight prior to use in experiments. Where indicated, ampicillin (100 µg/ml) or chloramphenicol (20 µg/ml, KIM D27 strains only) was added to media for selection of plasmids.

Primers used in this study are listed in Supplemental Table S5. Deletion of the open reading frame (ORF) of *yopK* in KIM D27 and CO92 was achieved by allelic exchange using the suicide vector pCVD442 and 1,000 bp upstream and downstream of *yopK* [36]. Candidates carrying the deletion of the *yopK* ORF were confirmed by PCR and Western blot and tested for the presence of all three virulence plasmids (pCD1, pMT1 and pPCP1; data not shown). *Y. pestis* CO92 pCD1^−^ was also generated using pCVD442, confirmed by PCR and found to be avirulent in a mouse model of bubonic plague (data not shown). Complementation of the *yopK* mutation in *Y. pestis* KIM D27 was achieved by amplifying the *yopK* promoter, open reading frame and putative transcriptional terminator from *Y. pestis* CO92 by PCR and cloning into a derivative of the low copy plasmid pHSG576 [59]. Restriction enzymes were purchased from New England Biolabs (Ipswich, MA). *E. coli* DH5α was generally used for cloning and S17-1λpir was used for propagating pCVD442 plasmids [60], [61].

### Construction of T45 and D46 point mutations

Mutations to residues T45 and D46 of YopK were created using PCR mutagenesis of the complementing plasmid (pDA15). Point mutations were introduced in primers that were used to amplify pDA15 with Phusion polymerase (New England Biolabs, Ipswich, MA). PCR products were gel purified using QIAquick Gel Extraction kit (Qiagen, Germantown, MD), followed by ligation and transformation into *E. coli*. Mutants were verified by DNA sequencing.

### Cell culture

RAW 264.7 or MH-S alveolar macrophages (American Type Culture Collection, Manassas, VA) were grown in DMEM with phenol red (Invitrogen, Carlsbad, CA) and 10% FBS (Hyclone, Logan, UT) or RPMI 1640 (Invitrogen, Carlsbad, CA) with 10% FBS and 0.05 mM 2-mercaptoethanol (Millipore, Billerica, MA), respectively. Culture media for propagating macrophages was supplemented with 1% penicillin/streptomycin (Invitrogen, Carlsbad, CA).

### Caspase-3 assay

Caspase-3 activity was assessed as previously described [62], [63]. Briefly, RAW 264.7 or MH-S (ATCC, Manassas, VA) cells (1×10^6^) were seeded in 12 well plates in DMEM with phenol red and 5% FBS and incubated overnight at 37°C with 5% CO_2_. *Y. pestis* strains were grown in HIB overnight, diluted 1∶10 in HIB containing 2.5 mM CaCl_2_ and grown 2 hours at 28°C. Cultures were shifted to 37°C for 1 hour, and added to RAW 264.7 or MH-S cells at a multiplicity of infection (MOI) of 20. Plates were centrifuged for 5 min at 41×g and incubated for 3.5 hours, or the indicated times, at 37°C and 5% CO_2_. After incubation, cells were harvested, lysed and processed using the EnzCheck Caspase-3 Assay Kit according to manufacturer's recommendations (Invitrogen, Carlsbad, CA).

Contribution of JNK, caspase-8, and caspase-9 to caspase-3 cleavage by *Y. pestis* KIM D27 and KIM D27 *yopK* was assessed using the assay described above. One hour prior to infection, fresh media was added to the wells containing the indicated inhibitor, either SP600125 (10 µM, Sigma Aldrich, St. Louis, MO), IETD (50 µM, Sigma Aldrich, St. Louis, MO), or Z-LEHD-FMK (50 µM, R & D Systems, Minneapolis, MN). Caspase-3 activation was detected using EnzChek (Invitrogen, Carlsbad, CA) according to the manufacturer's protocol; values were normalized to untreated cells infected with either KIM D27 or KIM D27 *yopK*.

### Cytotoxicity assay

Cytotoxicity was assessed by lactate dehydrogenase (LDH) release as previously described [62]. Briefly, cultures of *Y. pestis* strains were grown as described above. RAW 264.7 cells or MH-S cells (1×10^6^) were seeded in 12-well plates as described above and incubated overnight at 37°C, 5% CO_2_. Bacteria were added at an MOI of 20, plates centrifuged for 5 min at 41×g and incubated for 4 hours, or the indicated times, at 37°C, 5% CO_2_. Duplicate wells of macrophages that were not infected were then detergent lysed to determine the maximum amount of LDH signal; 100 µl of supernatant from each sample was removed and analyzed using the CytoTox-One Homogenous Membrane Integrity Assay according to manufacturer's instructions (Promega, Madison, WI). LDH activity was calculated as the amount present in the media divided by the maximum LDH released from detergent-lysed cells.

### Animals

#### Ethics Statement

These studies were carried out in strict accordance with the recommendations in the Guide for the Care and Use of Laboratory Animals of the National Institutes of Health and were approved by the Animal Care and Use Committee of the University of Missouri. All efforts were made to minimize suffering of the animals.

Male and female BALB/c or C57BL/6 mice, 6–8 weeks old, were purchased from Charles River Laboratories (Wilmington, MA) or Jackson Laboratories (Bar Harbor, ME), respectively. Male and female *Ccr2^−/−^* mice were purchased from Jackson Laboratories and bred and raised in barrier facilities at the University of Missouri. During pneumonic plague challenge, mice were maintained in select agent approved biosafety level 3 containment facilities at the University of Missouri. All infected mice were monitored regularly by weighing and assignment of health scores. Animals were euthanized by CO_2_ asphyxiation followed by bilateral pneumothorax or cervical dislocation, methods approved by the American Veterinary Medical Association Guidelines on Euthanasia. For challenge, mice were lightly anesthetized with isoflurane and 20 µl of the indicated dose was pipetted into the left naris. Following infection, animals were observed for up to 14 days for clinical signs of acute disease. The 50% lethal dose was calculated by the method of Reed and Muench [64].

### Histology

As indicated in the text, infected animals were euthanized, blood was collected by cardiac puncture, and organs removed and divided in half for histology and analysis of bacterial loads. Small lung lobes were analyzed for bacterial titer; the large lung lobe was inflated *in situ* with 10% formalin for histological analysis. All tissues were fixed for at least 96 hours and then stained with hematoxylin and eosin (H&E). Pathology was assessed by analysis by a veterinary pathologist and scored blindly for severity.

### Immunohistochemistry

Tissues that had been fixed in 10% formalin as described above were sectioned onto slides for immunohistochemical analysis. Slides were stained with anti-rat F4/80 (Serotec, Oxford, UK), anti-rat NIMP R14 (Ly6G/6C), (Santa Cruz Biotechnology, Santa Cruz, CA) , anti-rat caspase-3 (Trevigen, Gaithersberg, MD), anti-mouse CD3 (Dako, Carpinteria, CA), anti-mouse B220 (CD45R) (Trevigen, Gaithersberg, MD), or anti-mouse CCR2 (Abcam, Cambridge, MA) and detection was achieved by secondary staining with biotinylated rabbit anti-rat IgG and HRP-streptavidin (DAKO, Carpenteria, CA). Staining and detection were carried out according to the manufacturer's guidelines. For enumeration of caspase-3 positive alveolar macrophages, slides were blinded and 10 non-overlapping fields were assessed at 40× magnification.

### Flow cytometry analysis of pulmonary infiltrate

To analyze inflammatory cells in the lung, mice were euthanized at 24 HPI and the chest cavity was opened to expose the lungs and trachea. 0.5 ml of 2 mg/ml collagenase dispase (Roche Diagnostics, Indianapolis, IN) in 1× PBS (Gibco, Invitrogen, Carlsbad, CA) was injected through the trachea into the lungs with lungs remaining *in situ* for 3–5 minutes. Lungs were then removed from the mouse and homogenized in 1 ml additional collagenase dispase solution. Lung homogenate was incubated in collagenase dispase solution for 45 minutes at 37°C shaking at 150 rpm. After incubation, homogenate was strained through a 40 µm filter to remove tissue debris and red blood cells were lysed with a 1× ACK lysis solution for 90 seconds. Cells were resuspended in 100 µl FACS buffer (1× PBS with 1% FBS) for 20 minutes using TruStain (BioLegend, San Diego, CA) which blocks Fcγ receptor to prevent non-specific binding of antibody. Cells were then stained with anti-CD11c PE (BioLegend, San Diego, CA) at 1∶200 and anti-F4/80 FITC (BioLegend, San Diego, CA) at 1∶100. Cells were washed in FACS buffer and then fixed in 4% paraformaldehyde. Samples were run on a FACSCalibur (BD Biosciences, San Jose, CA) and analyzed with FlowJo software (FlowJo, Ashland, OR). Forward and side scatter were used to gate out cellular debris which typically includes dead cells and residual red blood cells.

### Enumeration of bacterial load

Following euthanasia, blood was collected and organs removed and divided in half as described above. For bacterial titers, tissues were homogenized in sterile PBS, serially diluted and plated in triplicate on HIA. Plates were incubated for 48–72 hours at 26°C.

### Serum cytokine analysis

Blood from BALB/c mice was collected at the indicated times post-infection and centrifuged to remove cells. Serum samples were stored frozen at −80°C until analyzed. Cytokine analysis was performed using ELISA or the Premix 13-plex kit (Millipore, Billerica, MA) according to manufacturer's instructions and analyzed by Illuminex using IS 100 software (Qiagen, Camarillo, CA).

### *In vitro* cytokine analysis

RAW 264.7 or MH-S cells (1×10^6^) were seeded in a 12-well plate as described above and incubated overnight at 37°C, 5% CO_2_. Cells were washed and fresh media was added containing 100 U/ml IFN-γ (Abcam, Cambridge, MA) followed by incubation for 4 hours prior to infection. Bacterial cultures were prepared as described above then added to macrophages at a MOI of 20; control cells were not infected. Plates were centrifuged at 41×g for 5 minutes at room temperature then incubated at 37°C, 5% CO_2_. After 8 hours, 1 ml of supernatant was removed and frozen at −80°C until analysis. Each supernatant was diluted 1∶10 in 1× assay diluent according to manufacturer's directions and analyzed for the presence of MCP-1 and IL-1β (BioLegend, San Diego, CA) by ELISA. Cytokine and chemokine concentrations were calculated according to manufacturer's directions using a standard curve.

### Statistical analysis

Data from all replicates were analyzed for statistical significance. Survival data were evaluated by the Gehan-Wilcoxon test; all other data were evaluated by Student *t*-test or one-way ANOVA with Tukey post-test using GraphPad Prism (GraphPad Software, La Jolla, CA).

## Supporting Information

Figure S1 ***Y. pestis*** **CO92** ***yopK*** **is severely attenuated for pneumonic plague.** BALB/c mice were challenged by intranasal infection of *Y. pestis* CO92 *yopK* at the indicated doses and (A) monitored for development of disease for 14 days (n = 10–12 mice, collected in two experiments). (B–C) Hematoxylin and eosin (H&E) stain of formalin-fixed lungs harvested from moribund mice infected with 1.8×10^7^ CFU *Y. pestis* CO92 *yopK*, time to acute disease was 2 days (n = 11 mice); (D–F) H&E stained lungs, liver and spleen from representative moribund mouse infected with 0.5× LD_50_ dose of CO92 *yopK*, time to acute disease was 12 days (n = 4, collected in three experiments). Boxes show 4× magnified section of indicated area.
(TIF)

Figure S2 **Deletion of** ***yopK*** **results in severe attenuation of bacterial growth in the spleen and liver.** BALB/c mice were challenged by intranasal infection with 1×10^4^ CFU of CO92 (A–B) or 1×10^6^ CFU CO92 *yopK* (C–D) and spleen (A,C) and liver (B,D) pathology were analyzed following H&E staining of formalin fixed tissue sections collected at 72 HPI. Images are representative of two experiments, n = 6 per group. Boxes show 4× magnified section of indicated area.
(TIF)

Figure S3 **Recruitment of monocytes, B and T cells occurs in the absence of YopK.** Tissue sections from 72 HPI from lungs of wild type mice challenged by intranasal infection with 1×10^4^ CFU wild type (A–D) or 1×10^6^ CFU *yopK* (E–H) *Y. pestis* CO92 were stained by immunohistochemistry with antibodies to NIMP R14 (A,E), F4/80 (B,F), CD3 (C,G), and B220 (D, H). Images are representative of 6 mice analyzed for each bacterial strain, collected in two independent experiments.
(TIF)

Figure S4 **Liver and spleen from mice infected with CO92 or CO92** ***yopK*** **show similar immune cell populations.** BALB/c mice were challenged by intranasal infection with 1×10^4^ CFU of wild type *Y. pestis* CO92 (A–H) or 1×10^6^ CFU Δ*yopK* (I–P) and euthanized at 72 HPI. Liver (E–H, M–P) and spleen (A–D, I–L) were analyzed for immune cell populations by immunohistochemistry for Ly6G/6C (A, E, I, M), F4/80 (B, F, J, N), CD3 (C, G, K, O), and B220 (D, H, L, P). Images shown are representative of two experiments, n = 6 mice per group.
(TIF)

Table S1 **Severity scoring of disease for moribund mice infected by** ***Y. pestis yopK*** **.**
(DOCX)

Table S2 **Pathology scores for intranasal infection of** ***Y. pestis*** **CO92** ***yopK*** **in BALB/c mice.**
(DOCX)

Table S3 **Serum cytokines found in wild type BALB/c mice following pulmonary infection.**
(DOCX)

Table S4 ***Y. pestis*** **and** ***E. coli*** **strains and plasmids used in this study.**
(DOCX)

Table S5 **Primers used in this study.**
(DOCX)
