# Supplementary figures and images for: Early Apoptosis of Macrophages Modulated by Injection of Yersinia pestis YopK Promotes Progression of Primary Pneumonic Plague
Source: PLoS Pathog. 2013 Apr 25;9(4):e1003324. doi: 10.1371/journal.ppat.1003324 (PMC3636031; doi:10.1371/journal.ppat.1003324)

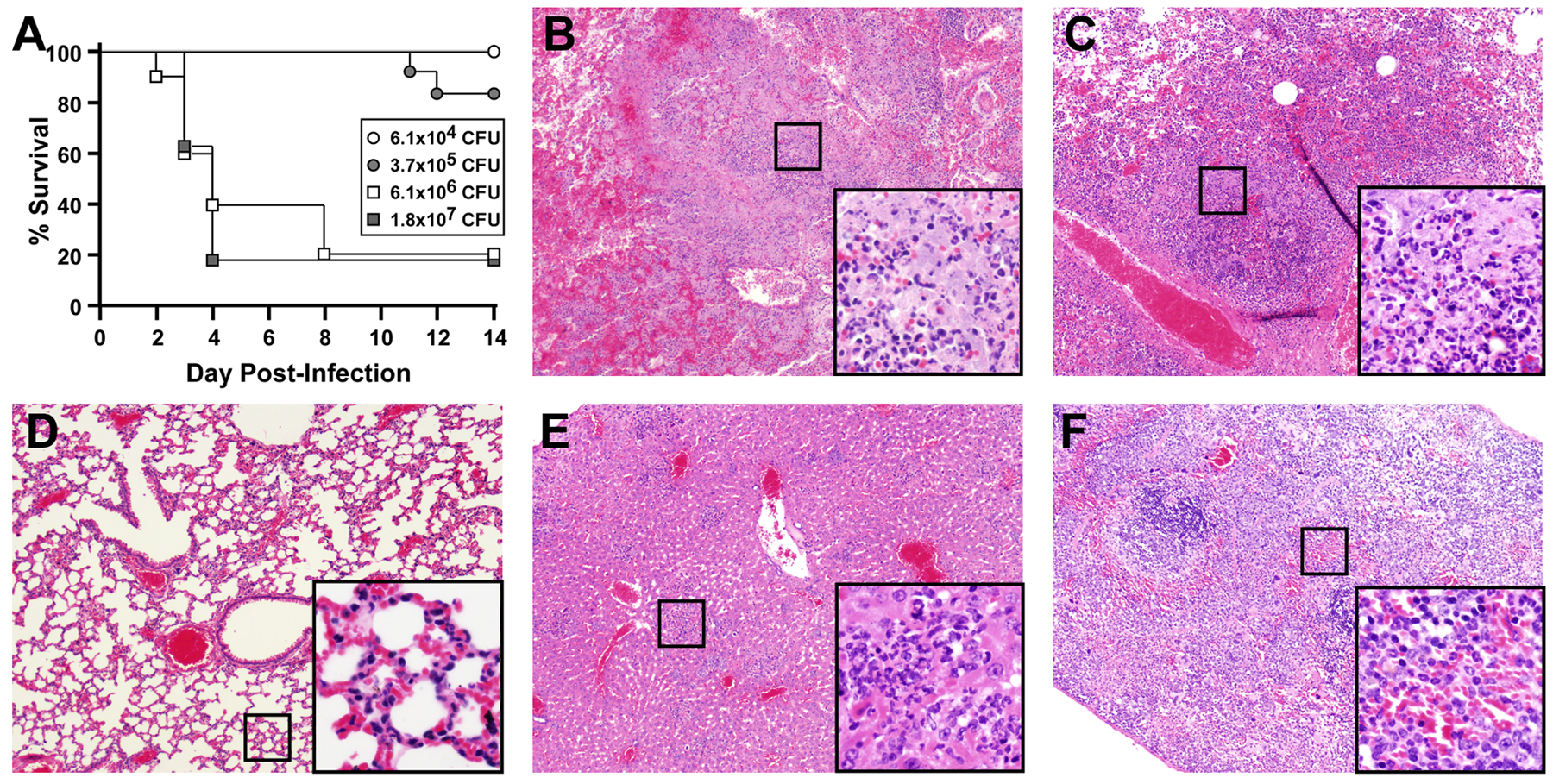

Supplement: Figure S1 — Y. pestis CO92 yopK is severely attenuated for pneumonic plague. BALB/c mice were challenged by intranasal infection of Y. pestis CO92 yopK at the indicated doses and (A) monitored for development of disease for 14 days (n = 10–12 mice, collected in two experiments). (B–C) Hematoxylin and eosin (H&E) stain of formalin-fixed lungs harvested from moribund mice infected with 1.8×10^7^ CFU Y. pestis CO92 yopK, time to acute disease was 2 days (n = 11 mice); (D–F) H&E stained lungs, liver and spleen from representative moribund mouse infected with 0.5× LD50 dose of CO92 yopK, time to acute disease was 12 days (n = 4, collected in three experiments). Boxes show 4× magnified section of indicated area. (TIF) [file ppat.1003324.s001.tif]

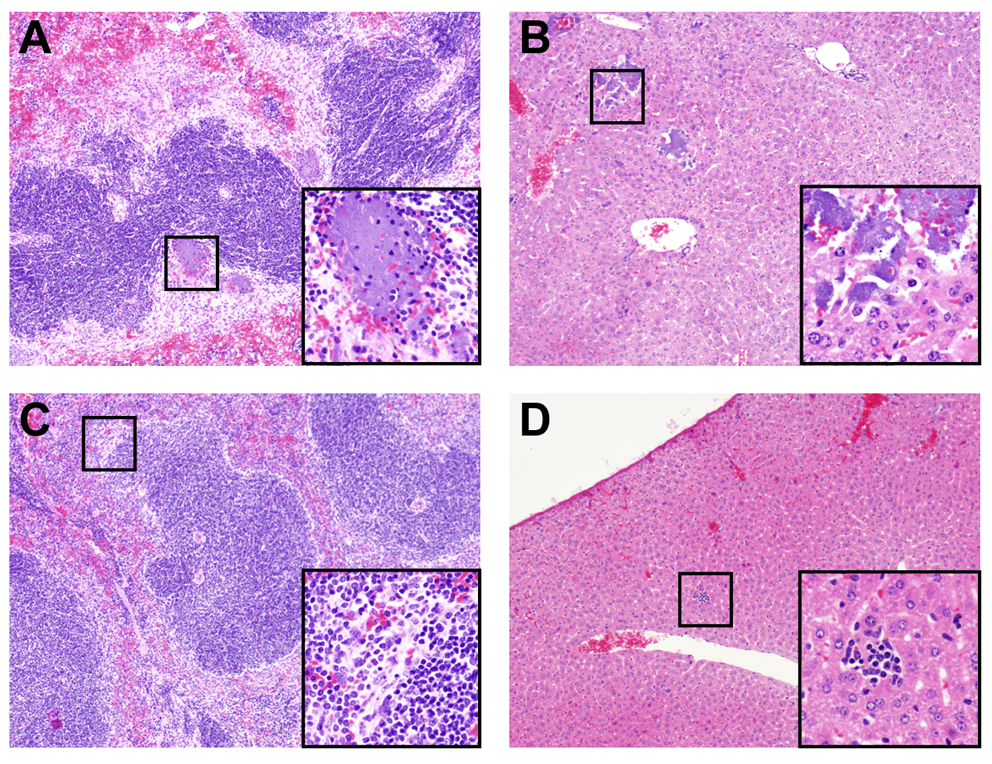

Supplement: Figure S2 — Deletion of yopK results in severe attenuation of bacterial growth in the spleen and liver. BALB/c mice were challenged by intranasal infection with 1×10^4^ CFU of CO92 (A–B) or 1×10^6^ CFU CO92 yopK (C–D) and spleen (A,C) and liver (B,D) pathology were analyzed following H&E staining of formalin fixed tissue sections collected at 72 HPI. Images are representative of two experiments, n = 6 per group. Boxes show 4× magnified section of indicated area. (TIF) [file ppat.1003324.s002.tif]

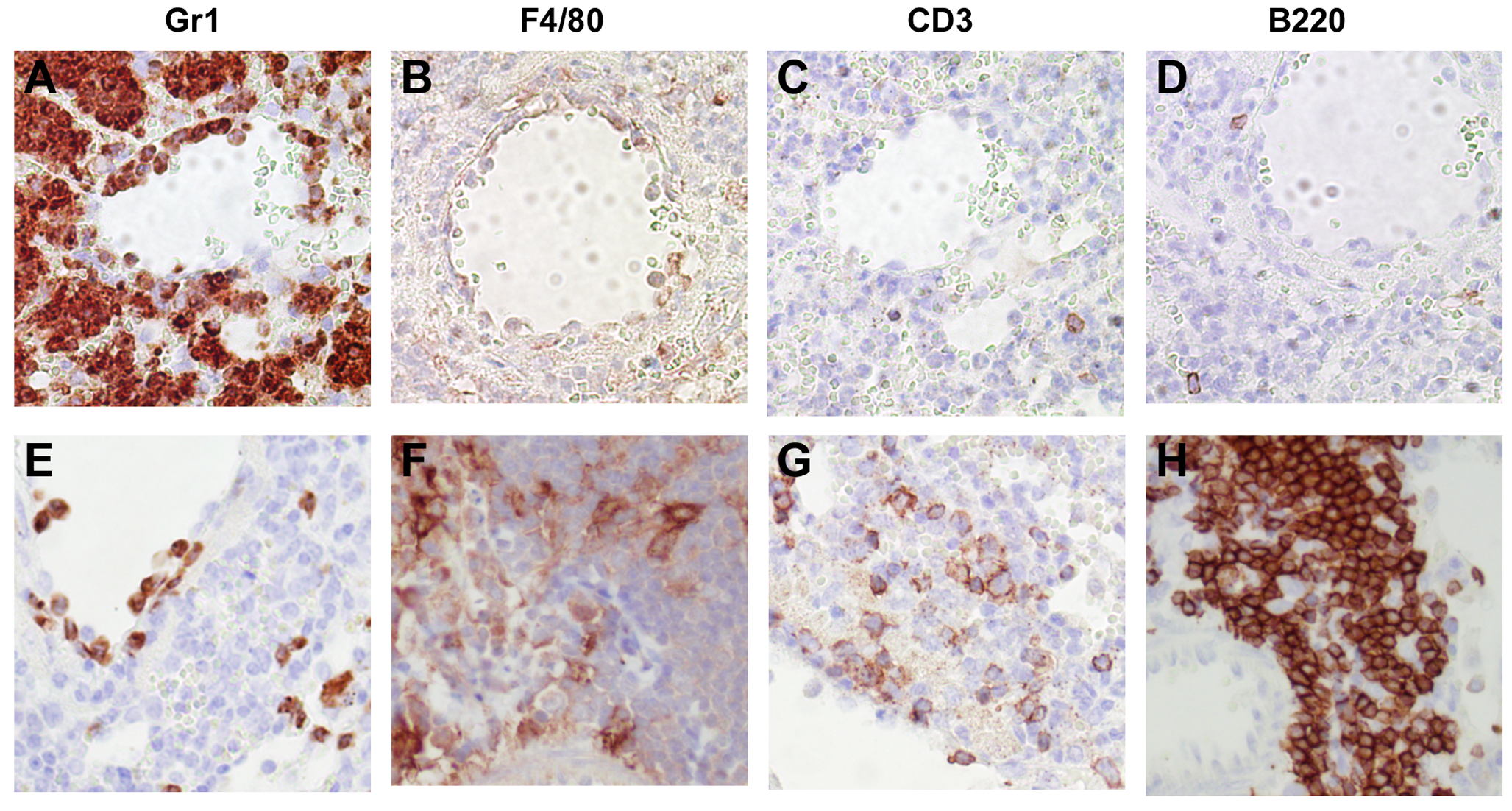

Supplement: Figure S3 — Recruitment of monocytes, B and T cells occurs in the absence of YopK. Tissue sections from 72 HPI from lungs of wild type mice challenged by intranasal infection with 1×10^4^ CFU wild type (A–D) or 1×10^6^ CFU yopK (E–H) Y. pestis CO92 were stained by immunohistochemistry with antibodies to NIMP R14 (A,E), F4/80 (B,F), CD3 (C,G), and B220 (D, H). Images are representative of 6 mice analyzed for each bacterial strain, collected in two independent experiments. (TIF) [file ppat.1003324.s003.tif]

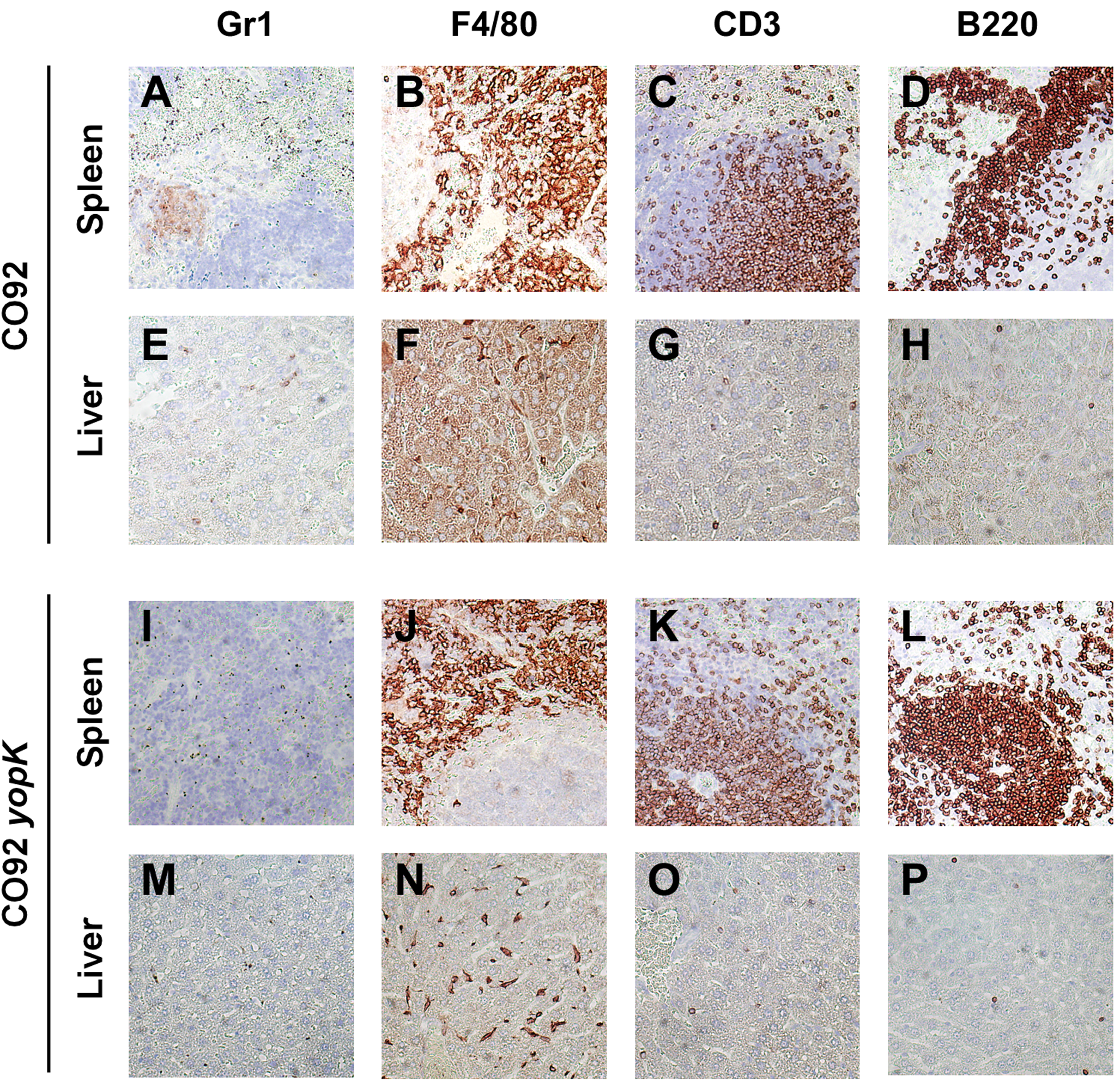

Supplement: Figure S4 — Liver and spleen from mice infected with CO92 or CO92 yopK show similar immune cell populations. BALB/c mice were challenged by intranasal infection with 1×10^4^ CFU of wild type Y. pestis CO92 (A–H) or 1×10^6^ CFU ΔyopK (I–P) and euthanized at 72 HPI. Liver (E–H, M–P) and spleen (A–D, I–L) were analyzed for immune cell populations by immunohistochemistry for Ly6G/6C (A, E, I, M), F4/80 (B, F, J, N), CD3 (C, G, K, O), and B220 (D, H, L, P). Images shown are representative of two experiments, n = 6 mice per group. (TIF) [file ppat.1003324.s004.tif]
