# Supplementary material for: Early Apoptosis of Macrophages Modulated by Injection of Yersinia pestis YopK Promotes Progression of Primary Pneumonic Plague
Source: PLoS Pathog. 2013 Apr 25;9(4):e1003324. doi: 10.1371/journal.ppat.1003324 (PMC3636031; doi:10.1371/journal.ppat.1003324)
Supplement: Table S1 — Severity scoring of disease for moribund mice infected by Y. pestis yopK . (DOCX) [file ppat.1003324.s005.docx]

Supplemental Table S1.

|  | **Lung** | | **Liver** | | **Spleen** | | **Blood** |  |
| --- | --- | --- | --- | --- | --- | --- | --- | --- |
| **Mouse^a^** | **Titer** | **Score^b^** | **Titer** | **Score** | **Titer** | **Score** | **Titer** | **TTD^c^** |
| **1** | ND^d^ | 2.6 | ND | 0.0 | ND | 0.3 | ND | 2 |
| **2** | ND | 0.4 | ND | 1.3 | ND | 2.0 | ND | 12 |
| **3** | ND | 2 | ND | 0.3 | ND | 0.8 | ND | 12 |
| **4** | 3.1x10^8^ | 0.2 | BD^e^ | 1.3 | BD | ND | BD | 10 |
| **5** | 3.3x10^4^ | 1.4 | BD | 0.3 | BD | 2.3 | BD | 8 |
| **6** | 3.73x10^6^ | 2.4 | 1.43x10^4^ | 0.0 | 2.23x10^3^ | 0.7 | 1.95x10^5^ | 8 |
| **7** | 1.00x10^3^ | 2 | 1.36x10^5^ | 1.3 | 2.00x10^2^ | 1.0 | BD | 14 |
| **8** | ND | 0.4 | ND | 1.3 | ND | ND | ND | 13 |

a: Total severity score as described in materials and methods; maximum score per tissue is 3.

b: mice infected with 7 x 10^5^ – 1.8 x 10^7^ CFU *Y. pestis* CO92 *yopK*; n=2

c: Time to death (days)

d: ND=not determined

e: BD=below detection limit (66 CFU)
