# Supplementary material for: Early Apoptosis of Macrophages Modulated by Injection of Yersinia pestis YopK Promotes Progression of Primary Pneumonic Plague
Source: PLoS Pathog. 2013 Apr 25;9(4):e1003324. doi: 10.1371/journal.ppat.1003324 (PMC3636031; doi:10.1371/journal.ppat.1003324)
Supplement: Table S2 — Pathology scores for intranasal infection of Y. pestis CO92 yopK in BALB/c mice. (DOCX) [file ppat.1003324.s006.docx]

Supplemental Table S2.

| **DPI^a^** | **Liver^b^** | | | **Spleen^b^** | | |
| --- | --- | --- | --- | --- | --- | --- |
|  | **Necrosis** | **Inflammatory Foci** | **Microgranuloma** | **Lymphocytolysis** | **Necrosis** | **Splenitis** |
| **2** | 0.0 | 0.0 | 1.3 | 0.0 | 0.0 | 0.0 |
| **4** | 0.3 | 0.5 | 1.7 | 0.0 | 0.0 | 0.0 |
| **6** | 0.0 | 0.0 | 1.0 | 0.2 | 0.0 | 0.0 |
| **8** | 0.0 | 0.0 | 1.0 | 0.8 | 0.0 | 0.0 |
| **10** | 0 | 0.2 | 0.7 | 0.7 | 0.0 | 0.0 |
| **12** | 0.0 | 0.0 | 0.3 | 1.3 | 0.0 | 0.0 |

a: DPI: Days post-infection

b: Mean severity score for group, maximum score for each criteria 3; n=3 per group.
