# Supplementary material for: Early Apoptosis of Macrophages Modulated by Injection of Yersinia pestis YopK Promotes Progression of Primary Pneumonic Plague
Source: PLoS Pathog. 2013 Apr 25;9(4):e1003324. doi: 10.1371/journal.ppat.1003324 (PMC3636031; doi:10.1371/journal.ppat.1003324)
Supplement: Table S3 — Serum cytokines found in wild type BALB/c mice following pulmonary infection. (DOCX) [file ppat.1003324.s007.docx]

Supplemental Table S3.

| Strain | Time | GM-CSF | TNFα | IL-2 | IL-4 | IL-5 | IL-7 | IL-12p70 | IL-13 |
| --- | --- | --- | --- | --- | --- | --- | --- | --- | --- |
| CO92^a^ | 6 | 20.67±22.44 | 0.29±0.71* | 0.20±0.32 | BD^c^ | 11.45±5.73 | 2.45±1.90 | 3.92±4.41 | 22.12±23.10 |
|  | 24 | 11.49±10.58 | 9.19±12.61 | 9.38±12.18 | 8.56±11.72 | 15.67±15.53 | 9.73±11.78 | 6.18±9.42 | 16.13±16.52 |
|  | 72 | 88.64±54.73* | 155.40±161.92 | 28.00±1.94* | 32.42±8.79* | 44.01±9.83* | 33.91±16.24* | 160.15±78.21* | 91.91±33.24* |
| *yopK*^b^ | 6 | 23.52±15.31 | 18.16±21.67 | 3.10±3.60 | BD | 8.45±6.59 | 7.79±6.13 | 10.26±8.78 | 14.38±5.23 |
|  | 24 | 9.42±13.72 | 10.25±11.46 | 5.09±8.57 | 4.44±9.92 | 10.25±12.06 | 7.09±8.10 | 18.71±10.92 | 15.61±13.11 |
|  | 72 | 15.05±14.57 | 1.32±1.73 | 0.58±0.89 | BD | 2.88±4.07 | 4.99±6.93 | 9.54±13.20 | 25.35±19.77 |

a: 1x10^4^ CFU intranasal challenge

b: 1x10^6^ CFU intranasal challenge

c: BD (Below Detection)

**p*<0.05 by unpaired Student *t*-test
