# Supplementary material for: Early Apoptosis of Macrophages Modulated by Injection of Yersinia pestis YopK Promotes Progression of Primary Pneumonic Plague
Source: PLoS Pathog. 2013 Apr 25;9(4):e1003324. doi: 10.1371/journal.ppat.1003324 (PMC3636031; doi:10.1371/journal.ppat.1003324)
Supplement: Table S4 — Y. pestis and E. coli strains and plasmids used in this study. (DOCX) [file ppat.1003324.s008.docx]

Supplemental Table S4.

| **Strain/Plasmid** | **Key Properties** | | **Reference** |
| --- | --- | --- | --- |
| ***E. coli* Strains** |  | |  |
| S17-1 | Sm^r^ Sp^r^ Tra^+^; *pro thi hsdR recA*; chromosomal integration of RP4-2-Tc::Mu-*Kan*::Tn*7* λ*pir*+ | | [[1](#_ENREF_1)] |
| DH5α | F^-^ , *lacZ*ΔαM15 *endA1 recA1 hsdR17*(r_M_- m_K_-) *supE44 thi-1 gyrA96* Δ(*lacZYA-argF*)U169 | | [[2](#_ENREF_2)] |
| ***Y. pestis* Strains** |  | |  |
| KIM5 | KIM6/pCD1Ap, pMT1^+^, pPCP1^+^, Δ*pgm,* Ap^r^ |  | [[3](#_ENREF_3)] |
| KIM5 *yopJC172A* | pCD1Ap *yopJC172A* (codon change of Cys172 to Ala172), Ap^r^ |  | [[3](#_ENREF_3)] |
| KIM D27 | KIM5 derivative, pCD1^+^, pMT1^+^, pPCP1^+^, Δ*pgm* |  | [[4](#_ENREF_4)] |
| KIM D27 *yopK* | KIM D27 derivative, with full deletion of *yopK* ORF |  | This Study |
| CO92 | pCD1^+^, pMT1^+^, pPCP1^+^, *pgm*^+^ |  | [[5](#_ENREF_5)] |
| CO92 *yopK* | CO92 derivative, with full deletion of *yopK* ORF |  | This Study |
| CO92 pCD1^-^ | pCD1^-^, pMT1^+^, pPCP^+^, Δ*pgm*^+^ |  | This Study |
| KIM6- | Derived from KIM6+, pCD1^-^, pMT1^+^, pPCP1^+^, Δ*pgm* |  | [[6](#_ENREF_6)] |
| **Plasmids** |  | |  |
| pCVD442 | Ap^r^, R6K *Ori* and *sacB* counter selection; suicide vector | | [[7](#_ENREF_7)] |
| pDA17 | pCVD442 containing sequence from 1000 bp upstream and 1000 bp downstream of the *yopK* ORF, used to delete the *yopK* ORF | | This Study |
| pDA15 | pHSG576 containing the *yopK* promoter, *yopK* ORF, and *yopK* terminator, used to complement *yopK* deletion, Cm^r^ | | This Study |
| pKP*yopK*02 | pDA15 *yopK* with codon change of thr45 to tyr45, Cm^r^ | | This Study |
| pKP*yopK*03 | pDA15 *yopK* with codon change of asp46 to lys46, Cm^r^ | | This Study |

**Supplemental References.**

1. Simon R, Priefer U, Pu¨hler A (1983) A broad host range mobilization system for *in vivo* genetic engineering: transposon mutagenesis in gram negative bacteria. Biotechnology 1.

2. Hanahan D (1985) DNA Cloning: A practical approach. In: Glover D, editor. McLean, VA: IRL Press. pp. 109.

3. Lilo S, Zheng Y, Bliska J (2008) Caspase-1 activation in macrophages infected with *Yersinia pestis* KIM requires the type III secretion system effector protein YopJ. Infect Immun 76: 3911-3923.

4. Brubaker R, Beesley E, Surgalla M (1965) *Pasteurella pestis*: Role of Pesticin I and iron in experimental plague. Science 149: 422-424.

5. Welkos S, Friedlander A, Davis K (1997) Studies on the role of plasminogen activator in systemic infection by virulent *Yersinia pestis* strain CO92. Microb Path 23: 211-223.

6. Fetherston J, Lillard JJ, Perry R (1995) Analysis of the pesticin receptor from *Yersinia pestis*: role in iron-deficient growth and possible regulation by its siderophore. J Bacteriol 177: 1824-1833.

7. Donnenberg M, Kaper J (1991) Construction of an *eae* deletion mutant of Enteropathogenic *Escherichia coli* by using a positive selection suicide vector. Infect Immun 59: 4310-4317.
