# Supplementary material for: Early Apoptosis of Macrophages Modulated by Injection of Yersinia pestis YopK Promotes Progression of Primary Pneumonic Plague
Source: PLoS Pathog. 2013 Apr 25;9(4):e1003324. doi: 10.1371/journal.ppat.1003324 (PMC3636031; doi:10.1371/journal.ppat.1003324)
Supplement: Table S5 — Primers used in this study. (DOCX) [file ppat.1003324.s009.docx]

Supplemental Table S5. Primers used in this study.

| **Gene of Interest** | **Primer Sequences** |
| --- | --- |
| *yopK* Flanking Regions | 5’ AAT CTA GAA TTG AGT AAA GTG TAT CG 3’  5’ AAG CAT GCT CCG TCA CCA TAG TAT AA 3’ |
| *yopK* ORF | 5’ AAC ATA TGT TTA TTA AAG ATA CTT ATA 3’  5’ AAG GAT CCT CAT CCC ATA ATA CAT TCT 3’ |
| *yopT* ORF | 5’ AAC ATA TGA ACA GTA TTC ACG GAG AC 3’  5’ AAG GAT CCT TAA ACC TCC TTG GAG TC 3’ |
| *yopM* ORF | 5’ AAC ATA TGT TCA TAA ATC CAA GAA A 3’  5’ AAG GAT CCC TAC TCA AAT ACA TGA TC 3’ |
| *caf1* ORF | 5’ AAC ATA TGA AAA AAA TCA GTT CCG T 3’  5’ AAG GAT CCT TAT TGG TTA GAT ACG G 3’ |
| *pla* ORF | 5’ AAA GGT ACC ATG AAG AAA AGT TC 3’  5’ AAA GGA TCC TCA GAA GCG ATA TTG CA 3’ |
| *yopK* Knockout Upstream | 5’ AAT CTA GAA TTG AGT AAA GTG TAT CGA TCT 3’  5’ AAG AAT TCA GTT ACT ACT CCC AAA TTT ACT T 3’ |
| *yopK* Knockout Downstream | 5’ AAG AAT TCT GAA GCT ATA TTA AAG AGT TTG G 3’  5’ AAG CAT GCT CCG TCA CCA TAG TAT AAT TGA 3’ |
| *yopK* Promoter | 5’ AAG AAT TCT CTG TAT AAC CTA TTT ATG TTA G 3’  5’ AAC ATA TGA GTT ACT ACT CCC AAA TTT A 3’ |
| *yopK* ORF | 5’ AAC ATA TGT TTA TTA AAG ATA CTT ATA ACA T 3’  5’ AAG GAT CCT CAT CCC ATA ATA CAT TCT TGA T 3’ |
| *yopK* Terminator | 5’ AAG GAT CCA GCT ATA TTA AAG AGT TTG GGA T 3’  5’ AAC TGC AGG TTT TTC TTC ATA TCA TCT TCC A 3’ |
| *yopK* T45Y Mutagenesis | 5’ GAT GTT TGT TCA TTG GT AAT 3’  5’ ATA TCT CAG TAA ATG AGC AGT 3’ |
| *yopK* D46K Mutagenesis | 5’ GTT TGT TCA TTG GTC AAT AGA G 3’  5’ TTT CGT TCT CAG TAA ATG AGC A 3’ |
